# 5D operando tomographic diffraction imaging of a catalyst bed

**DOI:** 10.1038/s41467-018-07046-8

**Published:** 2018-11-12

**Authors:** A. Vamvakeros, S. D. M. Jacques, M. Di Michiel, D. Matras, V. Middelkoop, I. Z. Ismagilov, E. V. Matus, V. V. Kuznetsov, J. Drnec, P. Senecal, A. M. Beale

**Affiliations:** 1 Department of Chemistry, University College London, 20 Gordon Street, London, WC1H 0AJ UK; 2 Research Complex at Harwell, Rutherford Appleton Laboratory, Harwell Science and Innovation Campus, Harwell, Didcot, OX11 0FA UK; 3 Finden Limited, Merchant House, 5 East St. Helens Street, Abingdon, OX14 5EG UK; 4 ESRF, 71 Avenue des Martyrs, 38000 Grenoble, France; 5 School of Materials, University of Manchester, Manchester, M13 9PL UK; 6 Flemish Institute for Technological Research, VITO NV, Boeretang 200, 2400 Mol, Belgium; 7 Boreskov Institute of Catalysis SB RAS, Pr. Akademika Lavrentieva 5, Novosibirsk, Russian Federation 630090

## Abstract

We report the results from the first 5D tomographic diffraction imaging experiment of a complex Ni–Pd/CeO_2_–ZrO_2_/Al_2_O_3_ catalyst used for methane reforming. This five-dimensional (three spatial, one scattering and one dimension to denote time/imposed state) approach enabled us to track the chemical evolution of many particles across the catalyst bed and relate these changes to the gas environment that the particles experience. Rietveld analysis of some 2 × 10^6^ diffraction patterns allowed us to extract heterogeneities in the catalyst from the Å to the nm and to the μm scale (3D maps corresponding to unit cell lattice parameters, crystallite sizes and phase distribution maps respectively) under different chemical environments. We are able to capture the evolution of the Ni-containing species and gain a more complete insight into the multiple roles of the CeO_2_-ZrO_2_ promoters and the reasons behind the partial deactivation of the catalyst during partial oxidation of methane.

## Abstract (web summary)

Multi-scale chemical imaging holds the potential to revolutionize our understanding of the relationships between structure and functionality in complex catalytic materials. Here the authors report the results from the first 5D tomographic diffraction imaging experiment of a complex Ni – Pd/ CeO_2_ – ZrO_2_/ Al_2_O_3_ catalyst used for methane reforming.

## Introduction

Heterogeneous functional materials and devices, like catalytic solids, batteries and fuel cells tend to possess complex structures where the 3D spatial distribution of the various components is rarely uniform^1–3^. Such materials are known to change with time under operating conditions, and in order to gain an insight into the structure–function relationships, it is highly desirable to study them in situ with spatially resolved techniques^4–7^. Non-destructive X-ray spectroscopic/scattering techniques are typically employed to study such materials, but it is the brilliant X-rays generated at synchrotrons coupled with state-of-the-art detectors and tomographic data collections that now allow the acquisition of spatially resolved signals from within the interiors of intact objects under operating conditions^8–11^. Operando chemical imaging in 5D by synchrotron X-ray Diffraction-Computed Tomography (XRD-CT) could emerge as a game-changing technique for the non-destructive investigation of functional materials in space and time under real process conditions^12–14^. This chemical tomographic technique, along with the complementary pair distribution function computed tomography technique, has been employed in several cases to study catalysts and more recently batteries, under in situ/operando conditions^15–24^. Such multi-scale chemical imaging tools hold the potential to revolutionise our understanding of the relationships between structure and functionality in complex, real world, catalytic materials (ie, in particular to better differentiate between reactive and spectator species); information that can help us unravel the mechanisms underpinning catalytic reactions and catalyst deactivation and rationally design improved materials.

The catalytic partial oxidation of methane (POX) is considered a very promising alternative to the highly energy-demanding steam reforming of methane (highly endothermic reaction) to produce synthesis gas (CO and H_2_) at gas-to-liquids (GTL) industrial plants (Fischer–Tropsch synthesis)^25–27^. The POX reaction is only mildly exothermic and it leads to a H_2_/CO molar ratio of 2, which is suitable for methanol and Fischer–Tropsch synthesis^28^. Given the promise of the POX reaction to decrease significantly the energy requirements of GTL plants, it is highly desirable to understand the spatio-temporal physico-chemical changes taking place in the real working catalyst. Such information is crucial in order to rationally design improved catalysts as the chemical (ie, gas composition) and temperature gradients in a reactor (both axially and radially) can have a direct impact on the chemical state of the catalyst^29–34^.

Ni/Al_2_O_3_-based catalysts have been the most widely studied POX catalysts mainly due to Ni being a cheap material compared to the noble metals (eg, Pd, Pt, Rh and Ru). However, Ni/Al_2_O_3_-based catalysts are usually prone to deactivation with time under POX reaction conditions for a variety of reasons; the most often reported in literature being carbon deposition on active Ni sites (metallic Ni being the active catalyst component), sintering of Ni particles and solid-state reactions involving Ni (eg, the formation of NiAl_2_O_4_ in Ni/Al_2_O_3_ catalysts)^35,36^. The CeO_2_–ZrO_2_ support promoters can offer improved oxygen storage capacity and redox properties, enhanced metal-support interaction (higher dispersion and stability of the Ni species) and increased catalytic performance at lower temperatures, while small amounts of noble metal (Pd, Pt, Rh and Ru) can enhance the reducibility of the Ni species through a hydrogen spillover mechanism^37–41^. In this work, we employed the XRD-CT technique to investigate the behaviour of a complex 10 wt.% Ni–0.2 wt.% Pd/10 wt.% CeO_2_–ZrO_2_/Al_2_O_3_ catalyst under different operating conditions.

Until now, the XRD-CT temporal resolution has been considered its main drawback along with problems associated with large datasets and high volume processing. However, in this work, we implemented a new data collection strategy, the concept of which we introduced previously, in principle applicable to all pencil beam tomographic techniques, where both tomographic axes (ie, translation and rotation) are allowed to move simultaneously^42^. This new data collection strategy, coupled with the brilliant X-rays produced at the ESRF and the state-of-the-art Pilatus2M CdTe area detector allowed us to collect an XRD-CT dataset in <2 min (ie, 117 s); the data collection rate is at least one order of magnitude faster than that previously reported (see also Supplementary notes 1 and 2). Also, we were able to meet the data handling challenge by analysing millions of diffraction patterns.

## Results

### Multi-length scale physico-chemical imaging

The in situ experiments were performed at beamlines ID31 and ID15A of the ESRF using the same catalyst (Supplementary Figures 1–3). The first experiment was a five-dimensional (5D) tomographic diffraction imaging experiment. Explicitly, here we mean three spatial dimensions, one diffraction dimension (*q*-space), and one dimension covering imposed chemical environment. Actually, one could consider this experiment covering more than five dimensions (ie, dependencies of multiple parameters such as phase distribution, crystallite size, and so on, over temperature and time too). The behaviour of a Ni–Pd/CeO_2_–ZrO_2_/Al_2_O_3_ catalyst was investigated during reduction and re-oxidation (redox experiment). In total, four 3D-XRD-CT datasets (each consisting of 30 XRD-CT datasets) were collected at the following operating conditions: ambient (1), 800 °C under He flow (2), 800 °C under 20% H_2_/He flow (3) and 800 °C under 20% O_2_/He flow (4). We present the results from the full Rietveld analysis of these datasets; the phase identification and Rietveld approach taken are described in the methods and Supplementary methods sections. The results represent the analysis of ~2 × 10^6^ diffraction patterns, which is an order magnitude larger compared to that previously reported^22,24,43–45^.

**Fig. 1:**
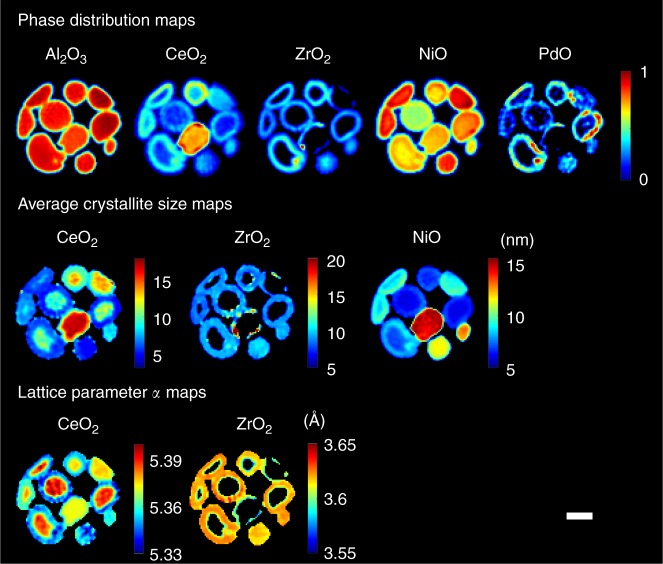
Maps derived from the Rietveld analysis of the XRD-CT data collected at the middle of the fresh catalyst bed. Top: phase distribution maps of Al_2_O_3_, CeO_2_, ZrO_2_, NiO and PdO corresponding to the normalised scale factors of these phases. Middle: average crystallite size maps of CeO_2_, ZrO_2_ and NiO (colorbar axes in nm). Bottom: Maps corresponding to the lattice parameter *a* of CeO_2_ and ZrO_2_ unit cells (colorbar axes in Å). Scale bar corresponds to 0.5 mm

The fresh catalyst is shown to contain crystalline NiO, PdO, CeO_2_, ZrO_2_ and Al_2_O_3_ (Supplementary Figure 4). Figure 1 presents the results from the XRD-CT dataset collected at the middle of the catalyst bed, the top row showing phase distribution maps of all the crystalline phases present in the catalyst particles. Each phase distribution map corresponds to the (normalised) values of the scale factors for each phase. The main catalyst support material, Al_2_O_3_, is seen to be homogeneously distributed over the catalyst particles and clearly defines the border and shape of each particle in the bed.

**Fig. 2:**
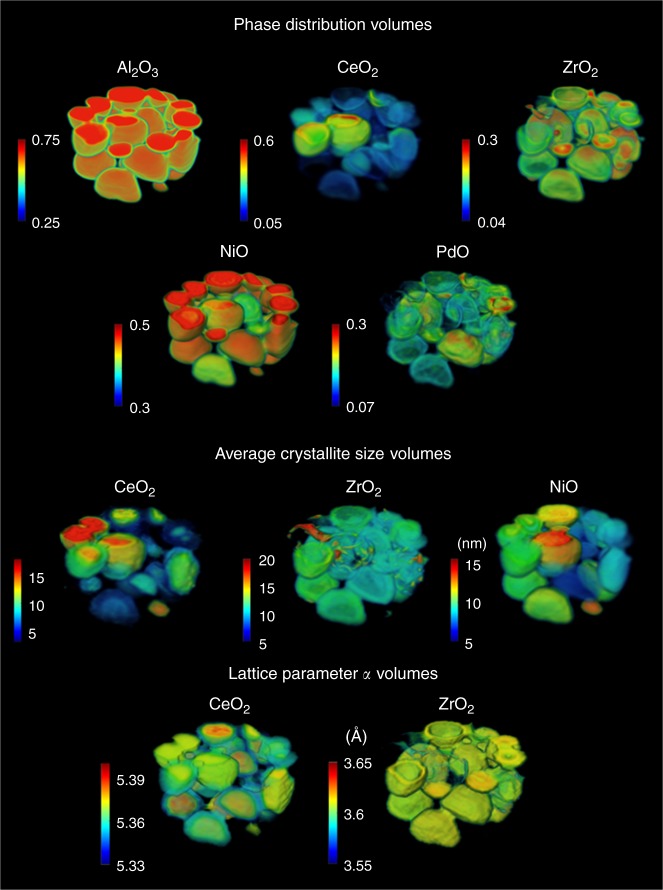
3D maps obtained from the Rietveld analysis of the 3D-XRD-CT data collected at room temperature. Top: volume rendering of the normalised scale factors (phase distribution volumes). The values in the colorbar axes have been chosen to achieve the best possible contrast. Middle: volume rendering of the average crystallite size maps of CeO_2_, ZrO_2_ and NiO (colorbar axes in nm). Bottom: volume rendering of the lattice parameter *a* of CeO_2_ and ZrO_2_ unit cells (colorbar axes in Å)

NiO is seen to be present in all catalyst particles as expected, due to the high Ni loading (10 wt.%), but its spatial distribution is not the same in all particles. We note, in the traditional 2D XRD-CT scan, it is not known whether the bottom, middle or top of each catalyst particle is probed, but as can be seen in Fig. 2, this is resolved in the 3D-XRD-CT; specifically, this figure shows the volume rendering of the scale factors of all phases. We see that NiO is found in higher concentration close to the surface of the catalyst particles. Similarly, PdO is also seen to be mainly present at/near the surface of the catalyst particles (this is clearly visible in Figs. 1 and 2), which is a direct outcome of the preparation method (impregnation) and the low Pd loading (0.2 wt.%). However, it can also be seen that there are regions where there is high concentration of PdO (hotspots in Fig. 1) indicating this phase is not well-dispersed over catalyst particles (see also Supplementary Figures 6–9).

The CeO_2_ and ZrO_2_ phases are most interesting. As is clear in Fig. 1, the ZrO_2_ map shows an egg-shell distribution (only present at/close to the surface of the catalyst particles). The same phenomenon is observed in the ZrO_2_ phase distribution volume shown in Fig. 2. Although one might expect the same distribution for the CeO_2_ phase (due to co-impregnation method used for the preparation of the CeO_2_–ZrO_2_/Al_2_O_3_ support), that is not the case. There is a strong signal generated by the CeO_2_ phase in the regions where ZrO_2_ is also present, but it does not diminish closer to the centre of the catalyst particles (as the ZrO_2_ signal does). As shown in Fig. 2, the observations regarding the CeO_2_/ZrO_2_ distributions are in full agreement with the rest of the XRD-CT datasets. The maps presented in the middle row of Fig. 1 correspond to values of the average crystallite size of the CeO_2_, ZrO_2_ and NiO phases, while the volume rendering of these data are presented in Fig. 2. It should be noted that the axial and radial gradients of these values (ie, spatial variations of the crystallite size values of each phase) provide the important physico-chemical information.

**Fig. 3:**
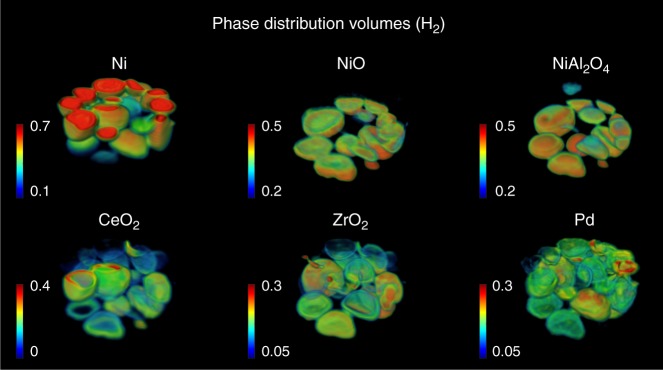
Catalyst activation process. Volume rendering of the normalised scale factors (phase distribution volumes) obtained from the Rietveld analysis of the 3D-XRD-CT data collected at 800 °C under 20% H_2_/He flow. The values in the colorbar axes have been chosen to achieve the best possible contrast

Characteristic examples are the crystallite size maps and volumes of the CeO_2_ and ZrO_2_ phases. The range of the ZrO_2_ crystallite size is generally seen to be very narrow (ca. 8–12 nm), but in the regions where there are hotspots (areas of high concentration) of ZrO_2_ (top row of Fig. 1), the average crystallite size increases significantly (middle row of Fig. 1). The same conclusion is reached by comparing the ZrO_2_ volumes shown in Figs. 2 and 3, as the regions of high ZrO_2_ concentration correspond to higher crystallite sizes. At first glance, one might think that the case of the CeO_2_ phase is similar (ie, due to the co-impregnation method). For example, the catalyst particle rich in CeO_2_ shown in Fig. 1 (approximately in the middle of the CeO_2_ phase distribution map), also corresponds to highly crystalline CeO_2_ (ie, highly crystalline compared to other regions of the sample). However, the range of CeO_2_ crystallite sizes is large in the other catalyst particles. In fact, the values of the CeO_2_ crystallite sizes are shown to follow an egg-yolk distribution. It can be seen in Fig. 1, that in the core of most particles, where there is no ZrO_2_, the CeO_2_ crystallite size varies between 8 and 12 nm, while at/close to the catalyst surface, where there is ZrO_2_ present_,_ the CeO_2_ crystallite size is less than half that value (ie, between 4 and 6 nm). This phenomenon is more apparent at the CeO_2_ crystallite size volumes presented in Fig. 2, where it can be readily seen that the CeO_2_ crystallite size follows an egg-yolk distribution in all catalyst particles.

The NiO crystallite size distribution shows a similar behaviour to that observed for the ZrO_2_ phase. Explicitly, the NiO concentration is relatively higher close to the surface of the catalyst particles. In these regions, the average NiO crystallite size is also typically higher compared to the inner core of the catalyst particles (Fig. 1). However, it should be noted that there is a peculiar case too. The catalyst particle in the middle of the catalyst bed in Fig. 1 shows the highest values for NiO crystallite size, but the NiO concentration is not higher compared to the other catalyst particles. It seems that the chemistry of this specific particle may differ from the rest as there is also high concentration of CeO_2_ (high with respect to the other catalyst particles). This may imply that in this particle there is a different chemical interaction between the various components (NiO, CeO_2_ and Al_2_O_3_). It should be noted though, that no other crystalline phases were identified to be present in this particle.

The maps shown in the bottom row of Fig. 2 correspond to the refined values of lattice parameter *a* of the CeO_2_ and ZrO_2_ unit cells. There are two distinct ranges regarding the values of the ZrO_2_ lattice parameter *a*. More specifically, as shown in Figs. 1 and 3, in the regions of the sample where there are high concentrations of ZrO_2_ (hotspots in Fig. 1 and regions of high intensity in Fig. 2), the values of the lattice parameter *a* are low (<3.6 Å). In all other regions, where CeO_2_ is present, the ZrO_2_ lattice parameter *a* corresponds to higher values (>3.62 Å). This implies that there are actually two different ZrO_2_ phases present in the sample: (1) a high-purity ZrO_2_ present in areas of high concentration of ZrO_2_ and (2) a Zr-rich Ce_x_Zr_y_O_2_ phase (Ce incorporation in the ZrO_2_ unit cell leads to larger unit cell)^46–49^. Interestingly though, two distinct CeO_2_ crystalline species seem to be present in the catalyst particles too. As shown in Figs. 1 and 3, the CeO_2_ lattice parameter *a* near the centre of the catalyst particles is notably higher (5.38–5.40 Å) compared to closer to the surface of the catalyst (5.34–5.36 Å), where ZrO_2_ is present too. This implies that there is incorporation of a Zr^4+^ substituent onto a Ce^4+^ site in the CeO_2_ unit cell in the regions where both CeO_2_ and ZrO_2_ are present leading to Ce-rich Ce_x_Zr_y_O_2_ phase (smaller unit cell compared to pure CeO_2_)^37–40^. Summarising the CeO_2_–ZrO_2_ results derived from the Rietveld analysis of the 3D-XRD-CT data of the fresh catalyst, it can be concluded that there are four distinct crystalline CeO_2_–ZrO_2_ species present in the catalyst (see also Supplementary Figure 10):

1. Small crystallites of a Ce-rich Ce_*x*_Zr_*y*_O_2_ (*x*»*y*) phase near the surface of the catalyst particles;
2. Larger crystallites of a higher purity CeO_2_ phase closer to the core of the catalyst particles;
3. Small crystallites of a Zr-rich Ce_*x*_Zr_*y*_O_2_ (*x*«*y*) phase near the catalyst surface;
4. Larger crystallites of a higher purity ZrO_2_ phase where there is high concentration of ZrO_2_ (near the surface of the catalyst particles—hotspots of this material).

### Catalyst activation and re-oxidation

After the 3D-XRD-CT measurement was performed at ambient conditions, the temperature of the system was increased to 800 °C (temperature ramp rate of 20 °C per min) under the flow of He (volumetric flow rate of 100 ml min^−1^). The catalyst remained at 800 °C under He flow (volumetric flow rate of 100 ml min^−1^) for 1 h while collecting a 3D-XRD-CT scan. The gas mixture was then switched to a 20% H_2_/He (total flow rate of 100 ml min^−1^). Another 3D-XRD-CT measurement was then performed under these reducing conditions in an attempt to capture the state of the activated catalyst. Finally, the gas mixture was switched to 20% O_2_/He (total flow rate of 100 ml min^−1^) and a last 3D-XRD-CT scan was performed (catalyst re-oxidation experiment).

The main difference in the state of the catalyst at 800 °C under He flow compared to the room temperature 3D-XRD-CT data is related to the Ni-containing phases. Specifically, we observe the presence of the undesired NiAl_2_O_4_ phase (Supplementary Figures 11 and 12). It has been previously reported in literature that crystalline NiAl_2_O_4_ can be observed in Ni/Al_2_O_3_ catalysts calcined at temperatures above 600 °C^50^. It is perhaps not surprising then that the NiAl_2_O_4_ phase is seen to be present in the catalyst at 800 °C^51^. This indicates that a reducing chemical environment (eg, H_2_) is essential in order to avoid the NiO to NiAl_2_O_4_ transition and reduce the NiO to the desired, active metallic Ni phase, as the formation and growth of the NiAl_2_O_4_ phase is a temperature-driven phenomenon taking place even under inert chemical environment (He flow). However, the phase distribution maps of NiO, NiAl_2_O_4_ and Ce_*x*_Zr_*y*_O_2_ revealed that NiO is mainly present near the surface of the catalyst particles (where the most of the Ce_*x*_Zr_*y*_O_2_ phases are present) while NiAl_2_O_4_ is closer to their core. This provides direct evidence that one of the roles of the Ce_*x*_Zr_*y*_O_2_ phases is to stabilise the NiO phase and suppress the formation of the undesired NiAl_2_O_4_ phase (Supplementary Figure 12).

**Fig. 4:**
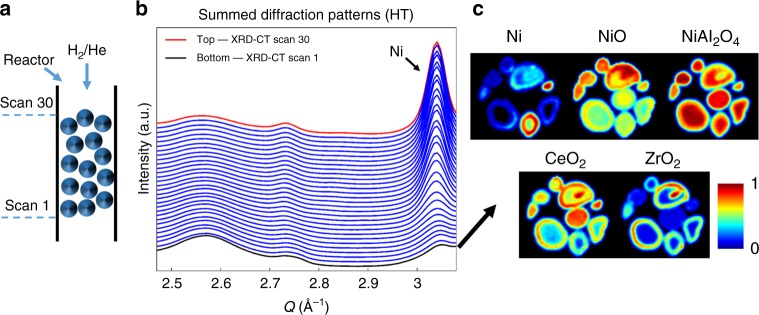
Reduction and activation of the catalyst bed. **a** Schematic representation illustrating the initial and final probing position during the 3D-XRD-CT at 800 °C under 20% H_2_/He flow. The gases were introduced into the reactor from the top as indicated by the blue arrows. **b** The summed diffraction patterns of the 3D-XRD-CT data (30 XRD-CT scans). The black arrow indicates the highest intensity Ni peak. **c** Phase distribution maps of Ni, NiO, NiAl_2_O_4_, CeO_2_ and ZrO_2_ corresponding to the normalised scale factors of these phases as derived from the Rietveld analysis of XRD-CT scan 1 as indicated by the black arrow

The results from the Rietveld analysis of the 3D-XRD-CT data collected during reduction (catalyst activation) are presented in Fig. 3. In contrast to the results obtained under He flow, there are significant differences between the 30 XRD-CT ‘slices’ consisting this 3D-XRD-CT dataset. This is clearly shown at the right side of Fig. 4 where the summed diffraction patterns from all 30 XRD-CT datasets collected during the 3D-XRD-CT measurement under reducing conditions are presented. The diffraction peak that appears at ca. *Q* = 3.04 Å^−1^ corresponds to the metallic Ni phase (reflection (111)). The crystalline metallic Ni phase is seen to form quickly and is present even in the first XRD-CT data collected under H_2_ flow (bottom of the sample volume probed—XRD-CT scan 1). However, the continuous growth of the Ni diffraction signal and decrease of the NiO and NiAl_2_O_4_ peaks (ca. *Q* = 3 Å^−1^ and *Q* = 2.55–2.65 Å^−1^, respectively) imply that NiO and NiAl_2_O_4_ were still present during the acquisition of this 3D-XRD-CT measurement (ie, in several XRD-CT scans). In order to decouple these phenomena, we performed another diffraction experiment where we show that the NiO/NiAl_2_O_4_/Ni concentration gradients shown in Fig. 4 are a purely temporal phenomenon (Supplementary Figures 13–15 and accompanying text). The retardation in the full reduction of the Ni–O and Ni–Al–O species could be due to the formation/presence of water produced from the formation of metallic Ni closer to the reactor inlet^19,23^.

The phase distribution volumes shown in Fig. 3 clearly demonstrate this Ni concentration gradient along the length of the catalyst bed. Similarly, the decrease of the concentration of the NiO and NiAl_2_O_4_ phases along the catalyst bed can also be readily observed. A closer inspection of the results also reveals that the NiAl_2_O_4_ phase, which is known to be more difficult to reduce compared to the NiO phase, remains present at positions of the catalyst bed where the NiO diffraction signal has already diminished^25,35^. Of course, as also shown in the 30 XRD-CT-summed diffraction patterns presented in Fig. 4, only metallic Ni is observed near the top of the catalyst bed.

The Ce_x_Zr_y_O_2_ mixed oxides seem to be the predominant phases compared to the respective higher purity CeO_2_ and ZrO_2_ phases. This is implied from the CeO_2_ phase distribution volume shown in Fig. 3 where CeO_2_ is seen to be mainly present near the surface of the catalyst particles, similar to the ZrO_2_ (ie, Zr-rich Ce_*x*_Zr_*y*_O_2_) phase distribution volume (egg-shell distribution). It should be emphasised that the most important result obtained from this 3D-XRD-CT dataset is related to the correlation between the Ni and the Ce_x_Zr_y_O_2_ phase distribution maps. Specifically, as shown on the right side of Fig. 4, the metallic Ni phase is first formed in the regions (initially following only an egg-shell distribution before being present everywhere in all catalyst particles) where the Ce_*x*_Zr_*y*_O_2_ phases are present. This result provides direct evidence that the most important role of the Ce_*x*_Zr_*y*_O_2_ promoters is to enhance the reducibility of the NiO and the formation of the active catalyst component, the metallic Ni phase.

Finally, it should be mentioned that metallic Pd is observed at the 3D-XRD-CT data collected under reducing conditions. The phase distribution volume of Pd shown in Fig. 3 is very well correlated with the PdO distribution presented previously in Fig. 2. It is also important to note that the metallic Pd peak (reflection (111)) is observed at larger *Q* values than expected (ca. *Q* = 2.85 Å^−1^) corresponding to a smaller unit cell than a pure Pd one (Supplementary Figure 17). This result implies that Ni is incorporated in the Pd unit cell (shift of diffraction peak to larger *Q* values) and that Pd exists as a Ni_*x*_Pd_*y*_ phase (alloy) rather than a high-purity Pd phase; the formation of Ni_*x*_Pd_*y*_ alloys has been previously reported in other catalytic systems^52–55^.

**Fig. 5:**
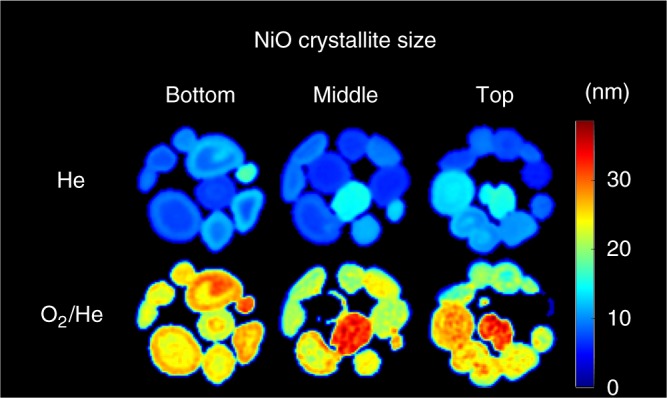
NiO crystallite size evolution. Maps of crystallite sizes of NiO (units in nm) as obtained from the Rietveld analysis of the XRD-CT data collected at the bottom, middle and top of the sample (XRD-CT scans 1, 15 and 30, respectively). Top row: catalyst under He flow, bottom row: catalyst under 20% O_2_/He flow. Scale bar corresponds to 1 mm

The last 3D-XRD-CT dataset was collected under the flow of 20% O_2_/He. The re-oxidation process of metallic Ni to NiO is really fast as the metallic Ni peak is absent in all 30 XRD-CT datasets and only the NiO and NiAl_2_O_4_ phases can be observed (Supplementary Figure 16). The most important results from this re-oxidation step are presented in Fig. 5 where the maps corresponding to the crystallite size of NiO, as obtained from the Rietveld analysis of the XRD-CT data collected at the bottom, middle and top of the sample under He and under 20% O_2_/He flow, are shown. It can be readily seen that the re-oxidation step at 800 °C led to a substantial increase in the crystallite size of the NiO phase, implying that sintering of the NiO species took place. In many regions of the sample, the NiO crystallite size more than doubled from ca. 5–15 nm to ca. 20–35 nm. The increase of the NiO crystallite size was not prevented even in the regions where the various CeO_2_/ZrO_2_/Ce_*x*_Zr_*y*_O_2_ promoters are present indicating that careful consideration must be given to the catalyst pre-treatment before switching to real reaction conditions.

**Fig. 6:**
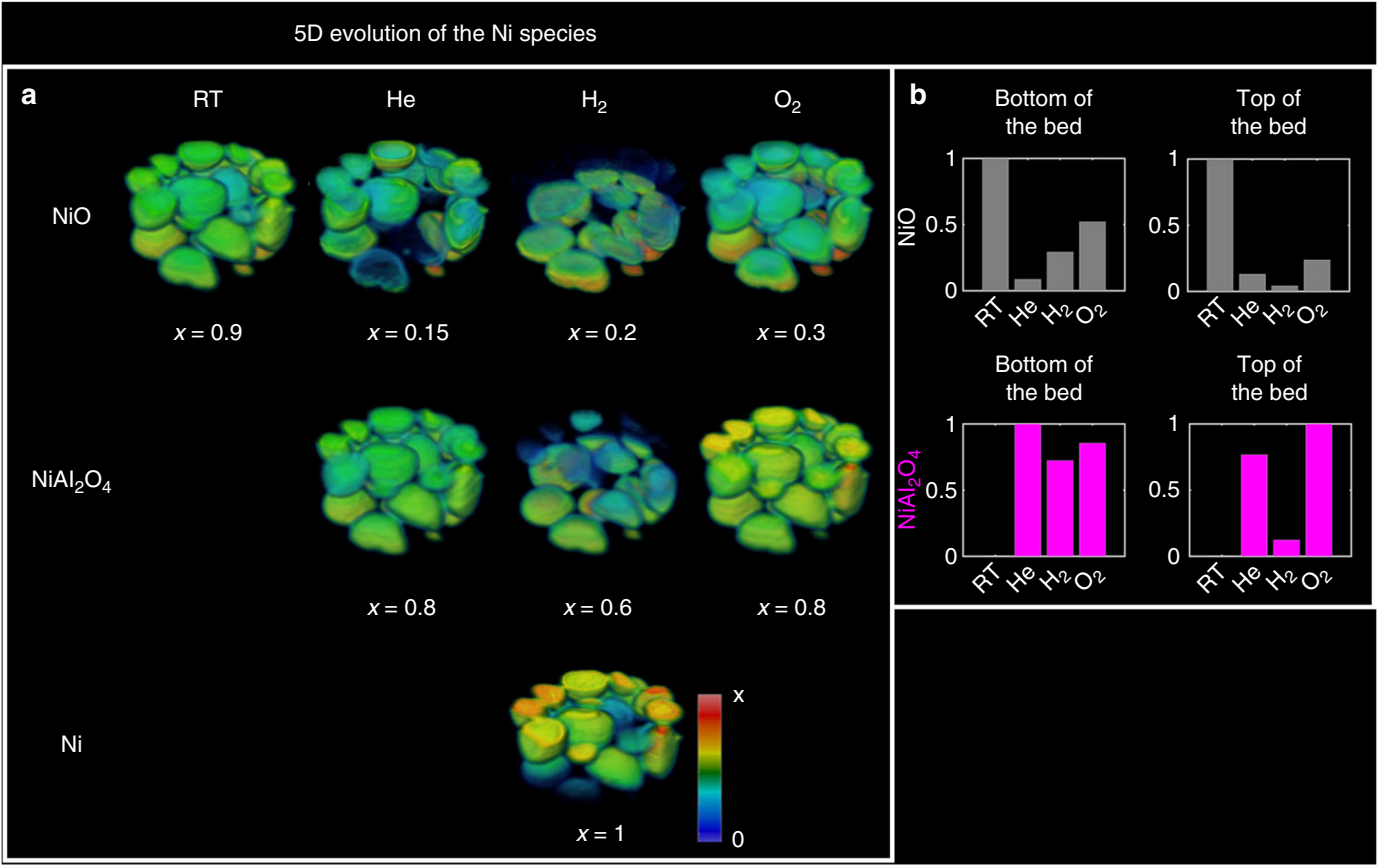
5D chemical evolution of the Ni-containing species in the catalyst bed. **a** Phase distribution volumes of NiO, NiAl_2_O_4_ and Ni as obtained from the Rietveld analysis of the 3D-XRD-CT data collected at the four different operating conditions. The values in the colorbar axes have been chosen to achieve the best possible contrast. **b** Solid-state evolution of the NiO and NiAl_2_O_4_ phases at the bottom and top of the catalyst bed during the redox experiment (ie, XRD-CT scan 1 and 30). The results presented in this figure correspond to the Rietveld analysis of ca. 1.2 × 10^6^ diffraction patterns

The evolution of the Ni-containing crystalline phases during this 5D tomographic diffraction imaging redox experiment is summarised in Fig. 6 where the phase distribution volumes of NiO, NiAl_2_O_4_ and Ni under the various operating conditions are displayed (panel a). As discussed previously, NiO is the only observed crystalline phase in the fresh catalyst, while NiAl_2_O_4_ is the predominant Ni-containing phase in the 3D-XRD-CT dataset collected at 800 °C under He flow. Under these conditions, the NiO follows mainly an egg-shell distribution, similar to the Ce_*x*_Zr_*y*_O_2_ phase (where *y* > *x*), while the NiAl_2_O_4_ phase is mainly present closer to the core of the catalyst particles. This information provides direct evidence that the Ce_*x*_Zr_*y*_O_2_ suppresses/delays the formation of the undesired NiAl_2_O_4_ phase.

As discussed previously, under reducing conditions, a Ni chemical gradient is observed along the length of the bed, which is a time effect (ie, delay in the full reduction of NiO and NiAl_2_O_4_). This is also illustrated in panel b where it is shown that first the NiO is reduced and then the NiAl_2_O_4_ phase (probing from bottom to top of the bed). A similar time effect is shown during the re-oxidation experiment as the top of the bed (last scan) contains less NiO compared to the bottom of the bed (first scan) indicating that the NiO to NiAl_2_O_4_ transition can also be captured.

### Partial oxidation of methane

The last experiment was an operando XRD-CT experiment of the same catalyst during the partial oxidation of methane. The same protocol was used for the pre-treatment of the catalyst as in the redox experiment. Specifically, the temperature of the system was increased under the flow of He up to 800 °C (temperature ramp rate of 20 °C per min) and then the catalyst was activated under the flow of 20% H_2_/He (total flow rate of 100 ml min^−1^) to form the metallic Ni phase. The catalyst bed was then exposed to a POX reaction mixture (ie, 90 ml min^−1^ of pure He, 12 ml min^−1^ of pure CH_4_ and 3 ml min^−1^ of pure O_2_—CH_4_/O_2_ molar ratio of 4:1), which was kept constant for the duration of the experiment and seven XRD-CT scans were collected in total.

**Fig. 7:**
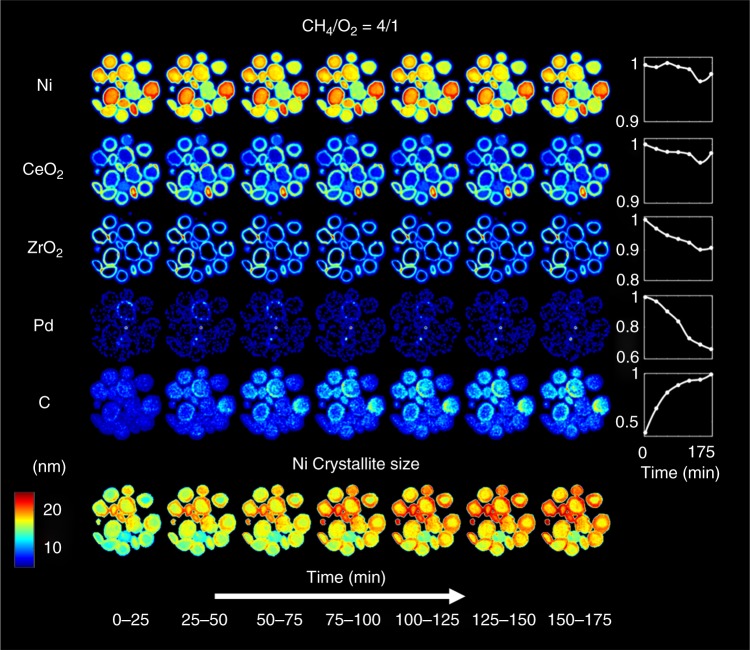
Chemical evolution of the catalyst during partial oxidation of methane. Phase distribution maps of Ni, CeO_2_, ZrO_2_, Pd and C (graphite) corresponding to the normalised scale factors of these phases derived from the Rietveld analysis of the XRD-CT data collected during the POX experiment. Bottom: maps of crystallite sizes of Ni under POX reaction conditions (colour bar axes in nm). Scale bar corresponds to 1 mm

In Fig. 7, the phase distribution maps of certain crystalline phases of interest, as obtained from the Rietveld analysis of the XRD-CT data collected at 800 °C under POX reaction conditions, are presented. These phase distribution maps correspond to the values of the scale factors for each phase normalised with respect to the maximum values. NiO and NiAl_2_O_4_ remain only in traces for the duration of the experiment, while Al_2_O_3_ is not changing; for these reasons, these maps are not shown here and emphasis is given on the active catalyst components. More importantly, the crystalline Ni phase is seen to be very stable for the duration of the POX experiment (ca. 3 h). We also note there is no evidence for formation of crystalline Ni carbide phases (eg, Ni_3_C or NiC)^56^.

The typical reaction mixture used to test the performance of POX catalysts is a gas mixture where the CH_4_/O_2_ molar ratio is 2:1 (stoichiometric ratio for the POX reaction)^57–59^. Here, a higher CH_4_/O_2_ molar ratio of 4:1 was used in an attempt to force the catalyst to deactivate faster and capture the corresponding solid-state changes. As it is shown in Fig. 7, the Ni remains in a metallic form for the duration of the experiment (ca. 3 h), which is an interesting result especially when one considers the methane-rich operating conditions. It should also be stated that no other crystalline Ni-containing phases were observed in the XRD-CT data beyond the trace amounts of NiO and NiAl_2_O_4_ mentioned previously. However, as is shown in the bottom part of Fig. 7, sintering of metallic Ni is seen to take place as a function of time during the POX experiment.

Figure 7 also tells us that the diffraction signal from both Ce_*x*_Zr_*y*_O_2_ phases is seen to decrease with time under reaction conditions although one may argue that the decrease is not very significant (ca. 10 % decrease after 3 h). The Pd phase (more precisely, the Ni_*x*_Pd_*y*_ alloy as discussed previously) is also seen to decrease as a function of time under the POX reaction conditions. It should be noted though that no other crystalline Pd-containing species were observed in the XRD-CT data collected during the POX experiment. The mass spectrometry data acquired during the POX experiment are presented in Supplementary Figure 18, serve to prove that the catalyst was captured in its active state during the POX reaction. No apparent deactivation of the catalyst was observed during the experiment, but as it is shown in Fig. 7, upon switching to the POX reaction mixture a new phase formed, which was identified as graphite (Supplementary Figure 17)^55^. From the phase distribution maps presented in Fig. 7, it can be clearly seen that the Ni and graphite maps are directly correlated. Specifically, the results suggest that the coke deposition initially takes place at the regions rich in Ni, which is the active catalyst component for the POX reaction. This graphite phase is seen to continuously grow during the POX reaction experiment. It is also implied that particles that are rich in Ce_*x*_Zr_1−*x*_O_2_ are less prone to graphite formation; in these particles, the Ce_*x*_Zr_1−x_O_2_ forms a protective layer near the surface of the catalyst particles. As mentioned previously, the POX reaction experiment lasted for ~3 h and it can be seen that the diffraction signal from this phase doubled during this time period (Fig. 7). It is reasonable to expect that the growth of graphite at the catalyst particles will probably influence the long-term performance of the catalyst (ie, by blocking the active Ni/Pd sites). This suggestion seems also to be consistent with laboratory catalytic activity measurements we have performed following the same catalyst pre-treatment and activation protocol (Supplementary Figures 19 and 20). Specifically, the catalyst showed high performance when it was exposed to POX reaction conditions (the CH_4_/O_2_ molar ratio of 2:1), leading to 84% CH_4_ conversion and almost 100% H_2_ yield. However, exposing the catalyst to the CH_4_/O_2_ molar ratio equal to 4:1 for 3 h, partially deactivated the catalyst as it did not regain its initial performance when the reaction mixture was switched back to 2:1. As shown by the in situ XRD-CT data, this is highly likely to be due to the graphite formation (see also Supplementary Figures 21–26).

## Discussion

It is well known that time-resolved in situ or operando X-ray spectroscopic or scattering studies provide information on evolving structure activity relationships in functional materials with researchers in the field of heterogeneous catalytic materials shown historically to be keen to exploit their potential^60^. In more recent times, traditional single-point measurements have been superseded (in terms of the information they provide) by X-ray chemical imaging studies and these have been shown to yield far greater insight into the structure of single particles or else a handful of particles in a cross-section^1,2,5,11,17,20,21^. Despite these important advantages, there will always be doubts as to whether a single particle/group of particles are truly representative of their ilk in terms of composition and/or behaviour (ie, in a reactor environment). The biggest challenge in this regard is to be able to follow changes in the catalyst particle structure as a function of their location in a reactor. It is well known that catalyst particles experience variation in reactant/product composition both radially and longitudinally, thereby rendering attempts to correlate structure–function relationships in a reactor difficult and in some cases impossible. The 3D chemical imaging results presented herein obtained in real time and under operando conditions therefore represent significant step in attempting to extract more meaningful correlations between structure and function in catalytic and other functional materials.

Specific to this study, we have shown how the Rietveld analysis of the reconstructed XRD-CT data allowed us to differentiate between four different Ce_*x*_Zr_*y*_O_2_ crystalline species present in the sample. This information could only be obtained by collecting the 3D-XRD-CT dataset and performing the Rietveld analysis. It should be emphasised that the analysis of the reconstructed data (>2 × 10^6^ diffraction patterns) although very challenging, is now possible on reasonable timescales so as to obtain unprecedented physico-chemical information regarding the state of the catalyst under different operating conditions. We were able to create, for the first time, 3D maps corresponding to phase distributions, crystallite sizes and unit cell lattice parameters, demonstrating the multi-length scale information that can be obtained from performing such an experiment and the corresponding data analysis. As such, we were able to effectively track the evolution of the crystalline Ni-containing species under different chemical environments (He, H_2_/He, O_2_/He, CH_4_/O_2_). NiO was the only crystalline Ni-containing phase present in the fresh catalyst and it was shown that a reducing environment is necessary in order to avoid the formation of the undesired NiAl_2_O_4_ phase at high temperatures. It was shown that the role of the Ce_*x*_Zr_*y*_O_2_ promoters is to suppress this conversion and enhance the reduction of the Ni oxides to the catalytically active metallic Ni. It should be emphasised that this chemical information could have not been obtained with any conventional technique but only from the spatially resolved diffraction signals present in an XRD-CT dataset.

The reduction process was seen to be significantly slower compared to the re-oxidation. Specifically, significant Ni/NiO/NiAl_2_O_4_ concentration gradients were observed along the catalyst bed during reduction but not during re-oxidation. This phenomenon was attributed to the water formation during the reduction of the NiO/NiAl_2_O_4_ species to metallic Ni. It was also shown that the re-oxidation of the catalyst at 800 °C under 20% O_2_/He flow can have a strong impact on the NiO crystallite size. Metallic Ni was seen to be the main crystalline Ni-containing phase of the catalyst under POX reaction conditions, but sintering of the Ni crystallites took place as a function of time under reaction conditions. More importantly though, crystalline graphite was seen to form and continuously grow under POX reaction conditions which, as the laboratory catalytic activity data collected under the same conditions indicate, caused the partial deactivation of the catalyst.

We have shown that it is possible to track the chemical evolution of many particles across a catalyst bed and relate these changes and variations to the non-uniform gas composition the catalyst particles experience. The insights and understanding gained from this in situ study can be used to rationally design improved methane-reforming catalysts. With the advancements in synchrotron brightness, detector performance, sample environment (new reactor cells) and data analysis (Rietveld analysis of XRD-CT data), multi-dimensional chemical imaging techniques are bound to become increasingly easier to perform and one can readily foresee that they will replace conventional in situ XRD and X-ray imaging as the preferred method for characterising functional materials and devices (eg, catalytic reactors, batteries and fuel cells).

## Methods

### Catalyst preparation

The 10 wt.% Ni–0.2 wt.% Pd/10 wt.% CeO_2_–ZrO_2_/Al_2_O_3_ catalyst was prepared by sequential impregnation method. The CeO_2_–ZrO_2_/Al_2_O_3_ support was prepared by the co-impregnation method^61,62^. The microspherical (γ + δ)-Al_2_O_3_ with granules size of ~500 μm was used. Approximately 500 µm of (γ + δ)-Al_2_O_3_ was impregnated by aqueous solution of salts (cerium nitrate Ce(NO)_3_ · 6H_2_O and oxychloride of zirconium ZrOCl · 8H_2_O) at the required ratio. The CeO_2_–ZrO_2_/Al_2_O_3_ was dried at 120 °C for 6 h and calcined under air at 850 °C for 6 h with a heating rate of 2 °C per min. The 10 wt.% CeO_2_–ZrO_2_/Al_2_O_3_ support was impregnated by aqueous solution of nickel nitrate salt Ni(NO_3_)_2_ · 6H_2_O of the appropriate concentration. Then, Ni/CeO_2_–ZrO_2_/Al_2_O_3_ was dried at 120 °C for 6 h and calcined in air at 500 °C for 4 h with a heating rate of 2 °C per min. The Ni/CeO_2_–ZrO_2_/Al_2_O_3_ was impregnated by aqueous solution of palladium nitrate Pd(NO_3_)_2_ salt of the appropriate concentration. The catalyst were then dried at 120 °C for 6 h and calcined in air at 500 °C for 4 h with a heating rate of 2 °C per min. The catalyst samples used in this study were kindly provided by the Boreskov Institute of Catalysis (BIC).

### Reactor cells

The three catalytic reactors investigated in this study consisted of 10 wt.%Ni–0.2 wt. Pd/10 wt.% CeO_2_–ZrO_2_/Al_2_O_3_ catalysts (ie, quartz capillary fixed bed reactors supported by glass wool); the catalyst loading was 35 mg for the operando experiment (4 mm outer diameter quartz capillary for the operando experiment, 2 mm for the 5D tomographic diffraction imaging experiment). In each case, the reactor was mounted into a gas delivery stub, itself mounted to a standard goniometer (to enable alignment). The goniometer was fixed to a rotation stage set upon a translation stage to facilitate the movements required for the CT measurement. At ID31, heating was achieved by virtue of two Cyberstar hot air blowers heating each side of the catalytic reactor while at ID15A a furnace designed for CT experiments was used. Temperature calibration was performed before all experiments using a thermocouple by measuring the temperature at the catalyst bed. During the operando XRD-CT measurements, the outflow gasses were monitored by mass spectrometry using an Ecosys portable mass spectrometer. The mass spec line was inserted inside the capillary from the top. The mass spec capillary is dragging with a constant flow rate of 20 ml min^−1^.

### 5D tomographic diffraction imaging measurements at ID31, ESRF

XRD-CT measurements were made at beamline station ID31 of the ESRF using a 70 keV monochromatic X-ray beam focused to have a spot size of 25 × 25 μm. 2D powder diffraction patterns were collected also using the state-of-the-art Pilatus3 X CdTe 2 M hybrid photon counting area detector. The total acquisition time per point was 15 ms (exposure time of 11 ms and readout time of 4 ms). Four 3D-XRD-CT scans of the Ni–Pd/CeO_2_–ZrO_2_/Al_2_O_3_ catalyst were acquired at different operating conditions: (1) at room temperature, (2) at 800 °C under He flow, (3) at 800 °C under 20% H_2_/He flow (reduction step) and (4) at 800 °C under 20% O_2_/He flow (re-oxidation step). Each 3D-XRD-CT scan composed of 30 XRD-CT scans, each one collected at a different vertical position (ie, 25 μm step size along the catalyst bed). Each XRD-CT scan lasted ~2 min. The tomographic measurements were made with 100 translation steps (translation step size of 25 μm) covering 0–180° angular range, in steps of 2.5° (ie, 72 line scans). The detector calibration was performed using a CeO_2_ NIST standard. Every 2D diffraction image was converted to a 1D powder diffraction pattern after applying an appropriate filter (ie, 1% trimmed mean filter) to remove outliers using in-house developed MATLAB scripts^63^. The final XRD-CT images (ie, reconstructed data volume) were reconstructed using the filtered back projection algorithm.

### In situ XRD mapping and XRD-CT measurements at ID15A, ESRF

XRD mapping and XRD-CT measurements were made at beamline station ID15A of the ESRF using a 91 keV monochromatic X-ray beam focused to have a spot size of 40 × 20 μm (horizontal × vertical). 2D powder diffraction patterns were also collected also using the state-of-the-art Pilatus3 X CdTe 2 M hybrid photon counting area detector. The total acquisition time per point was 10 ms. Initially, an XRD-CT scan was performed at the middle of the catalyst bed at ambient conditions. The XRD-CT scan was made with 151 translation steps (translation step size of 40 μm) covering 0–180° angular range, in steps of 1.44° (ie, 126 line scans). Calibration and processing (5% trimmed mean filter) was carried as described in the previous section (5D imaging measurements at ID31, ESRF) with X-ray detection again using the Pilatus3 X detector. The temperature of the system was increased under the flow of pure He (30 ml min^−1^) up to 800 °C with a ramp rate of 20 °C per min. An XRD-CT scan was collected and then the inlet gas mixture was switched to 20% H_2_/He (50 ml min^−1^). Eight successive XRD maps were collected covering the whole bed (151 translation steps and 12 *z* positions, each 0.5 mm apart). Each XRD map lasted ~5 min. Finally, a 3D-XRD-CT scan was collected after the XRD maps. The 3D-XRD-CT scan composed of 10 XRD-CT scans, each one collected at a different vertical position (ie, 80 μm step size along the catalyst bed).

### Operando XRD-CT measurements at ID31, ESRF

XRD-CT measurements were made at beamline station ID31 of the ESRF using a 70 keV monochromatic X-ray beam focused to have a spot size of 20 × 20 μm. Here, the total acquisition time per point was 20 ms. Tomographic measurements were made with 225 translation steps (translation step size of 20 μm) covering 0–180° angular range, in steps of 1.125° (ie, 160 line scans). Calibration and processing was carried as described in the section above (5D imaging measurements at ID31, ESRF) with X-ray detection again using the Pilatus3 X detector. The temperature of the reactor was then increased to 800 °C with a ramp rate of 20 °C per min under the flow of He (ie, 30 ml min^−1^). The inlet gas was then switched to a 20% H_2_/He gas mixture (total flow rate of 100 ml min^−1^). Finally, the catalyst bed was exposed to a POX reaction mixture (ie, 90 ml min^−1^ of He, 12 ml min^−1^ of CH_4_ and 3 ml min^−1^ of O_2_ having a CH_4_/O_2_ molar ratio of 4:1), which was kept constant for the duration of the experiment.

### Rietveld analysis of the XRD-CT data

Quantitative Rietveld refinement was performed using the reconstructed diffraction patterns using the TOPAS software, on a voxel by voxel basis^64^. The results from the refinements were imported into MALTAB in order to create the various figures presented in this work (eg, phase distribution maps based on the scale factors or weight percentages, lattice parameters, and so on). Unless stated otherwise, the Rietveld analysis of the XRD-CT data presented in this work was based on the intensity of the scale factors and should be treated as a semi-quantitative analysis. Rietveld analysis was performed using the summed diffraction pattern of each XRD-CT dataset prior to the Rietveld analysis of the XRD-CT data in order to have a good starting model before performing the batch Rietveld analysis.

## Electronic supplementary material

Description of Additional Supplementary Files

Peer Review File

Supplementary Information

Supplementary Movie 1

Supplementary Movie 2

Supplementary Movie 3

Supplementary Movie 4

## Data Availability

Copies of raw radially integrated XRD-CT data can be found at http://tiny.cc/NCOMMS18-12413A. All data are available from the corresponding authors on reasonable request.
