# Supplementary material for: 5D operando tomographic diffraction imaging of a catalyst bed
Source: Nat Commun. 2018 Nov 12;9:4751. doi: 10.1038/s41467-018-07046-8 (PMC6232103; doi:10.1038/s41467-018-07046-8)
Supplement: Supplementary file 1 — Description of Additional Supplementary Files [file 41467_2018_7046_MOESM1_ESM.pdf]

Description of Additional Supplementary Files

File Name: Supplementary Movie 1
Description: Video illustrating how 5D XRD-CT works

File Name: Supplementary Movie 2
Description: 3D dataset for catalyst before reaction

File Name: Supplementary Movie 3
Description: 3D dataset for catalyst after calcination

File Name: Supplementary Movie 4
Description: 3D dataset for detailing evolution of Ni containing phases
