# Supplementary material for: 5D operando tomographic diffraction imaging of a catalyst bed
Source: Nat Commun. 2018 Nov 12;9:4751. doi: 10.1038/s41467-018-07046-8 (PMC6232103; doi:10.1038/s41467-018-07046-8)
Supplement: Supplementary file 3 — Supplementary Information [file 41467_2018_7046_MOESM3_ESM.pdf]

Supplementary Information:

5D tomographic operando diffraction imaging of a catalyst bed

A. Vamvakeros, S. D. M. Jacques, M. Di Michiel, D. Matras, V. Middelkoop, I. Z. Ismagilov, E.V. Matus, V.V. Kuznetsov, J. Drnec, P. Senecal and A. M. Beale

Supplementary methods

Experimental setups at beamlines ID15A and ID31, ESRF

The experimental setups used at beamlines ID31 and ID15A for the high temperature XRD-CT experiments are shown in Supplementary Figures 1 ans 2 respectively. The fast XRD-CT data collection strategy involves continuous rotation of the sample under study. Unfortunately, this has implications when catalytic experiments are performed which require continuous flow of chemicals (reactants/products). Currently this means that traditional reactor cells for catalytic experiments cannot be used for fast XRD-CT measurements as the presence of gas lines (e.g. in heterogeneous catalysis experiments involving solid catalysts) typically limit the available angular range to 0 – 180 ° or best case scenario to 0 – 360 ° (i.e. for CT measurements). As a result, new reactor cells need to be designed that allow both free rotation of the sample and continuous flow of chemicals. In this experiment, this problem was overcome indirectly by inserting the gas line into the top of the cell but with no gas seal. This setup allowed both free rotation of the sample and the continuous flow of gases through the catalyst bed. Future experiments using this continuous rotation data collection strategy will make use of gas delivery through rotational slip rings.

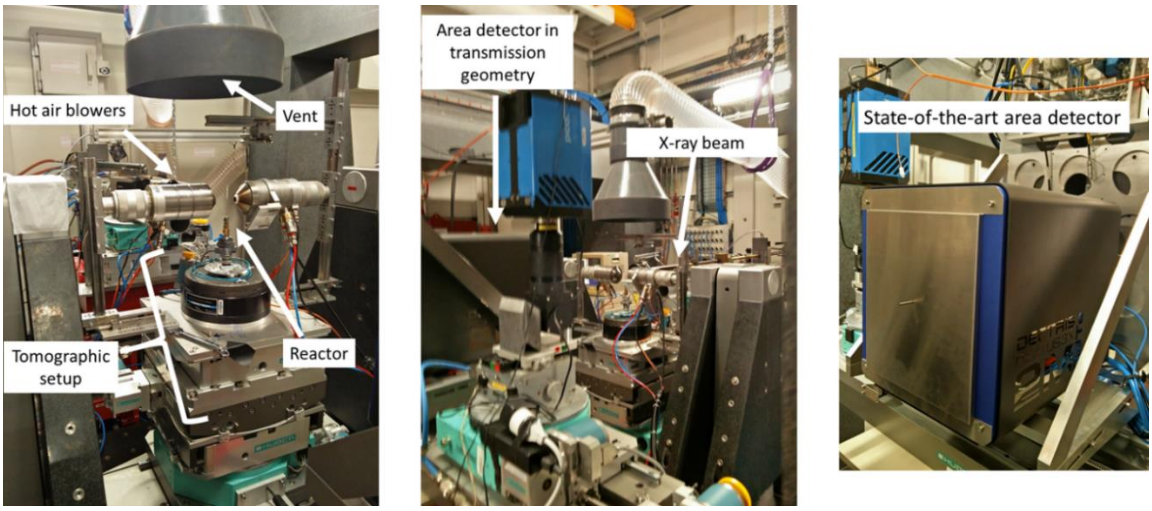

Supplementary Figure 1: Photographs of the ID31 XRD-CT experimental setup used at the ESRF. The reactor cell, tomographic setup, hot air blowers (heating system), vent and the Pilatus area detector are all indicated by the corresponding white arrows.

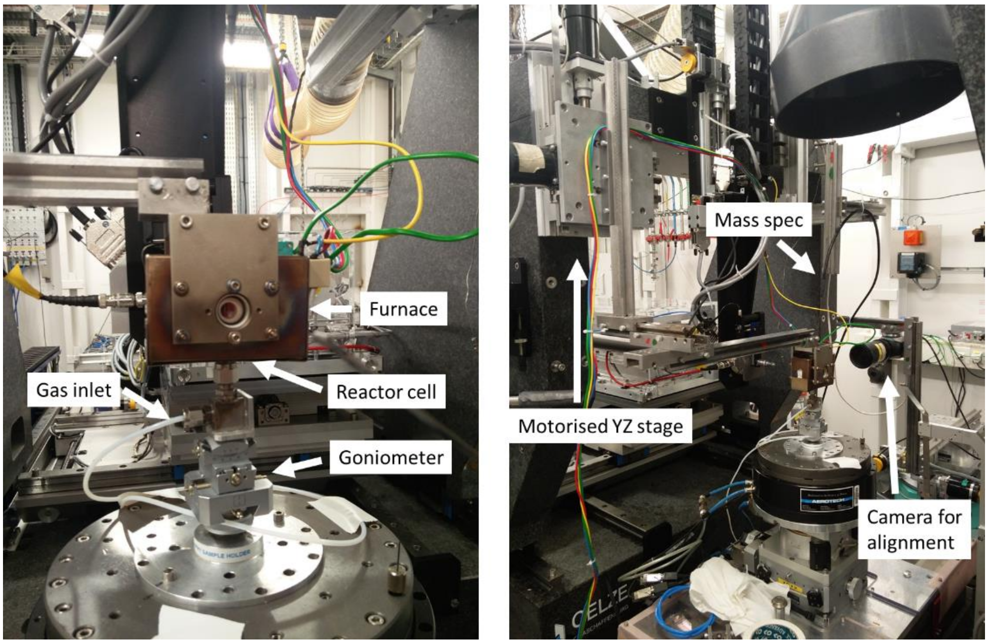

Supplementary Figure 2: Photographs of the ID15A XRD-CT experimental setup at the ESRF. The reactor cell, gas inlet, goniometer, furnace (heating system) and corresponding motorized YZ stage, mass spectrometer line and the camera used for the tomographic alignment are all indicated by the corresponding white arrows.

Temperature calibration was performed before all experiments using a thermocouple by measuring the temperature at the catalyst bed. The temperature calibration curve for the experimental setup with the two hot air blowers used at ID31 is shown in Supplementary Figure 3. The maximum temperature was used during the high temperature XRD-CT measurements (i.e. nominal 1000 °C and actual 800 °C). The temperature offset for the experimental setup with the furnace used at ID15A is minimal (i.e. nominal 825 °C and actual 800 °C).

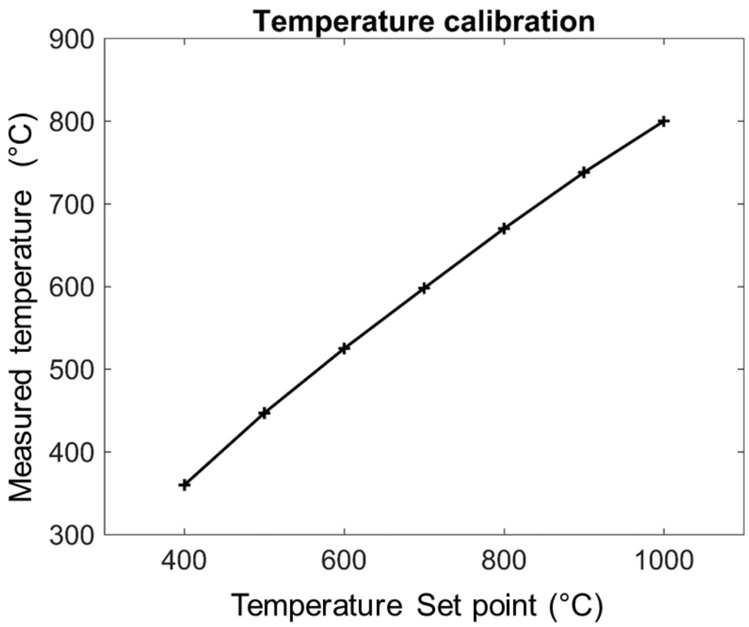

Supplementary Figure 3: Temperature calibration curve for the experimental setup with the two hot air blowers .

Supplementary note 1. Multi-dimensional chemical imaging

With the continuous advances in data acquisition strategies and methods/techniques development, it is becoming increasingly difficult to define the dimensions of in situ experiments. As such, the following tables serve as a guide to define the dimensions in multi-dimensional chemical imaging experiments. In Supplementary Table 1, the most common type of static scans (i.e. at ambient conditions/ the sample under study is not changing) are described. The conclusion is that for the type of scan (i.e. same number of spatial dimensions), in comparison to an absorption-contrast scan a chemical scan contains one extra dimension (i.e. the spectral or scattering dimension). For example, a static 3D-XRD-CT consists a 4D dataset (3D spatial and 1D scattering dimensions).

Supplementary Table 1: Type of static X-ray absorption-contrast and chemical imaging experiments

Type of scan	Acquired data	Spatial dimensions	Non-spatial dimensions	Overall dimensions
Point measurement	Intensity (0D)	0D	Static scan (0D)	0D
Linescan	"	1D	"	1D
Mapping	"	2D	"	2D
Tomography (2D)	"	2D	"	2D
Tomography (3D)	"	3D	"	3D
Point measurement	Spectrum or 1D diffraction/scattering pattern	0D	"	1D
Linescan	"	1D	"	2D

Mapping	"	2D	"	3D
Tomography (2D)	"	2D	"	3D
Tomography (3D)	"	3D	"	4D

The most common types of dynamic X-ray imaging experiments based on X-ray absorption-contrast are summarised in Supplementary Table 2. Each imposed operating condition acts as an extra dimension to the obtained data. For example, a series of conventional absorption-contrast 3D tomographic scans can yield a 4D dataset where the 4th dimension is time (if all other parameters are kept constant).

Supplementary Table 2: Type of dynamic X-ray imaging experiments based on X-ray absorption-contrast

Type of scan	Acquired data	Spatial dimensions	Non-spatial dimensions	Overall dimensions
Series of linescans	Intensity (0D)	1D	Temperature (1D), time (1D), chemical environment (1D), pressure (1D), potential/current (1D)	2D to nD (n > 2)
Series of maps or 2D tomograms	"	2D	"	3D to nD (n > 3)
Series of 3D tomograms	"	3D	"	4D to nD (n > 4)

Similar to the aforementioned approach, a series of 3D-XRD-CT scans yield, in the simplest case scenario, a 5D dataset (3D spatial, 1D scattering and 1D temporal dimensions). However, if other operating conditions are changed, then it can yield an multi-dimensional dataset (e..g temperature (1D), chemical environment (1D), pressure (1D)).

Supplementary Table 3: Type of dynamic X-ray chemical imaging experiments

Type of scan	Acquired data	Spatial dimensions	Non-spatial dimensions	Overall dimensions
Series of linescans	Spectrum or 1D diffraction/scattering pattern	1D	Temperature (1D), time (1D), chemical environment (1D), pressure (1D), potential/current (1D)	3D to nD (n > 3)
Series of maps or 2D tomograms	"	2D	"	4D to nD (n > 4)
Series of 3D tomograms	"	3D	"	5D to nD (n > 5)

Supplementary note 2. Fast XRD-CT and comparison with literature

In 1987, Harding et al. were the first to introduce a new materials characterization technique termed X-ray diffraction computed tomography (XRD-CT).[1] XRD-CT couples traditional powder X-ray diffraction with first generation (i.e. pencil beam approach) computed tomography (CT). It was first demonstrated using a lab diffractometer and more than a decade later, in 1998, Kleuker et al. were the first to implement XRD-CT using synchrotron light for medical imaging applications (soft tissue).[2] The total acquisition time of that XRD-CT scan (using an 80 keV monochromatic pencil beam) was 2 h for 100 × 100 pixels reconstructed images using an area detector (900 × 900 μm^2 beam size using slit systems). XRD-CT was re-introduced a decade later, in 2008, by Stock et al. and Bleuet et al.[3, 4] Stock et al. also used a ca. 80 keV monochromatic pencil beam but with a smaller beam size of 100 × 100 μm^2 acquiring in total 714 powder diffraction images. It should be mentioned that the acquisition time per point (exposure plus readout time) was 10 s. Bleuet et al. used an 18 keV monochromatic pencil beam with a beam size of 1.6 × 2.3 μm^2 (vertical × horizontal) but the experimental details regarding the tomographic scan (i.e. number of translations and projections) and the acquisition time per point were omitted in the paper (it was only claimed that a 100 × 100 × 100 μm^3 data volume with a voxel size of 3 μm could be achieved in 6 h). Since then, XRD-CT has gained a lot of attention as a chemical imaging technique and has been used to study numerous different systems including biological samples, automotive paints, cement, radioactive samples and batteries among others.[5, 6, 7, 8, 9, 10, 11, 12, 13, 14, 15, 16, 17, 18, 19] In the aforementioned studies, XRD-CT was employed either to study static samples or for ex situ characterization of functional materials (e.g. catalysts and batteries). However, it is generally accepted in literature that functional materials can change under working conditions; there are several review papers emphasizing the need for in situ/ operando techniques to characterize catalytic and electrochemical systems under real working conditions in order to gain a proper understanding of structure-activity relationships.[20, 21, 22, 23, 24, 25, 26, 27, 28, 29, 30, 31] XRD-CT has recently been exploited as a chemical imaging tool to study several catalytic systems under real process conditions and in all cases new physico-chemical information was revealed due to the spatially-resolved signals obtained from such measurements; information that is/can be lost in bulk measurements.[32, 33, 34, 35, 36] However, the temporal resolution of the XRD-CT technique has been always considered to be its main drawback. This is clearly implied in Supplementary Table 4 where the experimental details of most XRD-CT studies conducted in the past decade are presented (i.e. from papers where information available). It can be seen that in most studies, the total acquisition time of an XRD-CT scan is in the order of several hours which is far from ideal for dynamic experiments.

Supplementary Table 4: Experimental details of XRD-CT studies performed during the 2008-2016 period. CS: Current Study

**Energy (keV)**	**Beam size (μm × μm)**	**Number of diffraction patterns**	**Acquisition time per point (ms)**	**Total acquisition time (min)**	**Year**	**Ref**
80.715	100 × 100	714	10000	660-720	2008	[3]
30	15 × 15	4941	10000	-	2009	[5]
18	2 × 4	7500	-	480	2010	[6]
18	2 × 4	6480	800	480	2010	[7]
86.88	100 × 100	1290	400	10	2011	[32]
29	0.3 × 0.3	8000	5000	720	2011	[8]
69.77	50 × 100	4800	-	17	2012	[33]
65	15 × 150	8190	1200-1300	-	2012	[10]
17	2 × 5	13189	1000	540	2012	[19]
86.7	150 × 150	8000	150	25	2013	[12]
86.88	100 × 100	1290	400	10	2014	[34]
29.6	0.2 × 0.11	22806	1000	-	2014	[13]
13	2 × 2	1517	2000	120	2015	[35]
89.965	100 × 100	2500	1000	420	2015	[15]
93	25 × 25	14700	50	40	2015	[36]
65	25 × 25	25200	1000	540	2016	[17]
75	25 × 25	7200	15	<2	2018	CS
95	45 × 20	4617	11	<1	2018	CS

The chosen acquisition time per point (ATPP) during an XRD-CT scan is based on the quality of the powder diffraction images obtained (i.e. user's decision during the experiment). This means that the ATPP does not depend only on the number of photons arriving at the sample per unit of time (synchrotron/beamline specific and X-ray optics dependant) but also on the detector capabilities. Nowadays, state-of-the-art single photon counting detectors (e.g. PILATUS CdTe series) are considered to be noise-free allowing very fast acquisition times. For example, it is now considered trivial to perform XRD-CT experiments at the high energy beamlines of the ESRF (e.g. ID11, ID15, ID31) with ATPP of 10s of ms which is orders of magnitude faster compared to what was feasible till recently (i.e. ATPP of 100s of ms or several s).[36, 37] However, there is also another equally

important factor which contributes to the time required to perform an XRD-CT scan and it is has not been investigated/discussed in literature in the past. This is the dead time of the measurement which depends not only on the motors' capabilities (fast motors are essential for fast XRD-CT scans) but also on the chosen data collection strategy. We recently provided a short review on the available data collection strategies and also introduced a new one, termed as interlaced XRD-CT, which allows, post experiment, choice between temporal and spatial resolution.[37] Herein, we present a new data collection strategy which minimizes the dead time during an XRD-CT scan. In fact, the total acquisition time of such an XRD-CT measurement can be calculated by just multiplying the ATPP with the total number of diffraction patterns collected. The breakthrough in this ultra-fast XRD-CT data collection strategy lies on the fact that both the rotation and the translation axis are continuously moving during the acquisition of the tomographic scan.

In our first attempt to perform the fast XRD-CT scan at beamline ID31 of the ESRF, the ATPP was 15 ms (11 ms exposure time and 4 ms readout time). When these fast XRD-CT experiments were performed, the newly purchased Pilatus3 X CdTe 2M detector had not been fully integrated with the beamline hardware. More specifically, there was a limitation associated with writing and transferring data from the detector PC to the beamline PCs (i.e. network limitation). However, even with these limitations, the total acquisition time of each XRD-CT scan was less than 2 min (108 s to collect 7200 images) which is a technical breakthrough when compared to what was feasible in the past (Table 4). These limitations had been overcome when we performed the static ultra-fast 3D-XRD-CT scan of the fresh catalyst at beamline ID15A. The 3D-XRD-CT scan composed of 80 XRD-CT scans. Each XRD-CT scan lasted less than 1 min (57 s). The total acquisition time per point was 11 ms (exposure time of 10 ms and readout time of 1 ms). The tomographic measurements were made with 81 translation steps (translation step size of 35 μm) covering an 0 – 180 ° angular range with 57 angles (4617 images per XRD-CT dataset). This consists both the largest 3D-XRD-CT volume and the shortest time needed to complete a single XRD-CT scan (Supplementary Figure 6). This technical achievement is a milestone towards real-time 3D chemical imaging of materials.

Supplementary discussion

Phase identification results of the fresh catalyst

As mentioned in the main paper, each 3D-XRD-CT dataset during the 5D redox experiment composed of 30 XRD-CT scans performed at different positions along the catalyst bed (z step size of 25 μm). The phase identification of the fresh catalyst was performed using the summed diffraction pattern from the XRD-CT scan collected at the middle of the 3D stack (XRD-CT scan 15). The

crystalline phases identified were: CeO_2 (ICSD: 72155), ZrO_2 (ICSD: 66781), NiO (ICSD: 9866), PdO (ICSD: 24692) and theta-Al_2O_3.[4, 25, 38] The results of the phase identification are presented in Supplementary Figure 4 where a region of interest of the summed diffraction pattern is shown. In the interest of clarity, only the high intensity peaks of Al_2O_3 are presented (theta-Al_2O_3 corresponds to a low symmetry, monoclinic unit cell generating numerous diffraction peaks). It should be noted that $NiAl_2O_4$ was not detected on the fresh catalyst. These phases have been identified in ex situ studies of similar catalytic systems.[39, 40, 41, 42, 43, 44, 45]

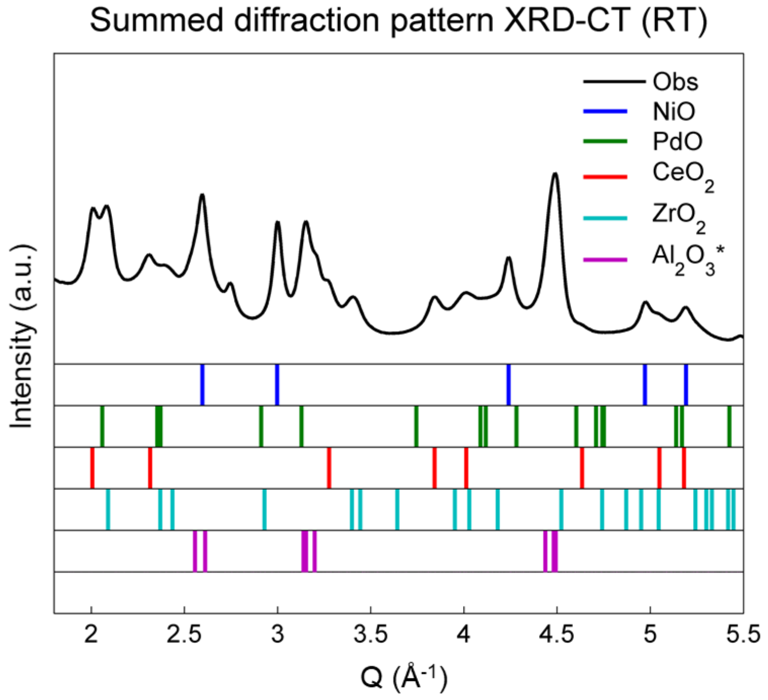

Supplementary Figure 4: Phase identification of the 10 % Ni-0.2 % Pd/10 % CeO_2-ZrO_2/Al_2O_3 catalyst. Black line: the summed diffraction pattern from the room temperature XRD-CT scan (i.e. after applying a binary mask to the reconstructed data in order to extract the diffraction patterns generated only by the sample), Blue ticks: NiO, Green ticks: PdO, Red ticks: CeO_2, Cyan ticks: ZrO_2, Magenta ticks: Al_2O_3 (high intensity peaks only)

Rietveld analysis of the XRD-CT data

An instrument parameter file was created from information derived from Rietveld analysis of CeO_2 diffraction data collected during the beamtime. A Chebyshev polynomial was used to fit the background and a pseudo-Voigt profile function to fit the diffraction peaks.[46] A CeO_2 CIF file was obtained from the ICSD database to be used for the Rietveld refinement (ICSD: 72155). This instrument parameter file was then used for the Rietveld analysis of the XRD-CT data. The same data processing protocol was followed for all the XRD-CT data mentioned in this work. The results from the quantitative Rietveld refinement using the summed diffraction pattern of the XRD-CT data collected at room temperature are shown in Supplementary Figure 5 (Rw = 0.026 on 1500 observations).

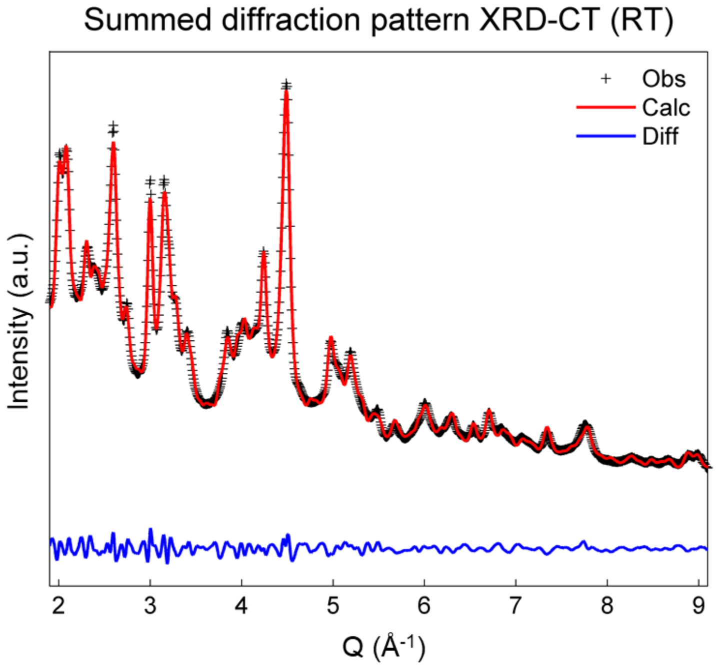

Supplementary Figure 5: Fresh Ni-Pd/CeO_2-ZrO_2/Al_2O_3 catalyst. Quantitative Rietveld refinement using the summed diffraction pattern of a room temperature XRD-CT dataset (XRD-CT scan 15).

Prior to the Rietveld analysis of the reconstructed data (4D matrix in the case of 3D-XRD-CT data), the reconstructed XRD-CT images were processed using the images corresponding to the high intensity peak/peaks of Al_2O_3 (ca. Q = 4.5 $Å^{-1}$). More specifically, masks (binary images) were created after performing image segmentation of the Al_2O_3 images by thresholding, in order to mask out the SiO_2 capillary (reactor vessel) and the empty space between the catalyst particles. The Rietveld analysis of the acquired XRD-CT data was then performed, after cropping appropriately the reconstructed data, on a line-by-line basis using the TOPAS software (all the powder diffraction patterns present in each row of each XRD-CT dataset were processed simultaneously).[47] These pre-processing steps were performed in order to minimize the required time for the Rietveld analysis of the XRD-CT data (i.e. number of diffraction patterns to be processed). Apart from refining the background and the scale factors of all crystalline phases, the crystallite size of each phase was calculated too. Finally, the unit cell parameters of CeO_2 and ZrO_2 were added to the refinement as a crude inspection of the reconstructed data prior to the Rietveld analysis revealed that there were significant variations in the unit cell parameters of CeO_2 and ZrO_2 over the sample (spatial variations). That was not the case for the PdO and the Al_2O_3 unit cell parameters which were not refined. From a materials chemistry perspective, there are not expected to be significant variations in the unit cell parameters of the Al_2O_3 it being the support of the catalyst. Additionally, refining the low symmetry Al_2O_3 unit cell parameters (lattice parameters a, b, c and β angle) does not only significantly increase the required computational time to perform the Rietveld analysis but can also

lead to a less stable refinement (higher number of parameters being refined simultaneously). Regarding the PdO, only the high intensity diffraction peak (ca. Q = 2.35 $Å^{-1}$ corresponding to (002) and (011) reflections) is observed in some regions of the sample and as it is shown in Supplementary Figure 5 there are other crystalline phases generating diffraction peaks in that Q region (i.e. CeO_2, ZrO_2 and Al_2O_3). Of course, it is also expected that there should not be significant variations in the PdO unit cell parameters as the 3D-XRD-CT measurement was performed at room temperature. As a result, it was considered prudent to not refine the lattice parameters of PdO and Al_2O_3.

X-ray micro CT and sub-minute XRD-CT measurements at ID15A, ESRF

X-ray micro-CT measurements were performed at beamline ID15A of the ESRF using a 42 keV monochromatic X-ray beam. Radiographs were recorded with an X-ray imaging camera (CCD) and the pixel resolution was 3.18 μm. Each micro-CT scan consisted of 1900 projections (radiographs) covering an angular range of 0 – 190 ° (i.e. angular step size of 0.1 °). Flat-field and dark current images were also collected prior to the micro-CT measurements and were used to normalize the acquired radiographs before the tomographic reconstruction. The tomographic data were reconstructed using the filtered back projection algorithm.

Ultra-fast XRD-CT measurements were also performed at beamline station ID15A of the ESRF using a 95 keV monochromatic X-ray beam focused to have a spot size of 45 μm x 20 μm. 2D powder diffraction patterns were collected using the Pilatus3 X CdTe 2M area detector. The 3D-XRD-CT scan composed of 80 XRD-CT scans, each one collected at a different vertical position (i.e. 50 μm step size along the catalyst bed). Each XRD-CT scan lasted less than 1 min (57 s). The total acquisition time per point was 11 ms (exposure time of 10 ms and readout time of 1 ms). The tomographic measurements were made with 81 translation steps (translation step size of 35 μm) covering an 0 – 180 ° angular range with 57 angles. The detector calibration was performed using a CeO_2 NIST standard. Every 2D diffraction image was converted to a 1D powder diffraction pattern after applying an appropriate filter (i.e. 5 % trimmed mean filter) to remove outliers using pyFAI.[48, 49] The final XRD-CT images (i.e. reconstructed data volume) were reconstructed using the filtered back projection algorithm.

The phase distribution volumes presented in Supplementary Figure 6 correspond to the volume rendering of the scale factors for each crystalline phase, as obtained from the Rietveld analysis of the ultra-fast 3D-XRD-CT data of the fresh catalyst. Each of these data volumes was normalised with respect to the maximum intensity. As expected, these results are in full agreement with the results presented in Figures 1 and 2 of the main paper. Al_2O_3 being the main support of the catalyst, is seen to be homogeneously present in all catalyst particles. On the other hand, the ZrO_2 phase is seen to follow an egg-shell distribution. The CeO_2 phase is also seen to be predominantly

present near the surface of the catalyst particles but there are also numerous particles where it is present near the core of the particles too. The NiO phase is present in all catalyst particles but it can also be seen that not in the same amount. Finally, the PdO phase is seen to follow a similar distribution to the ZrO_2 phase but it can also be clearly seen that there are region of very high concentration of this phase (i.e. hotspots). This indicates that the Pd species are not well distributed over the catalyst particles.

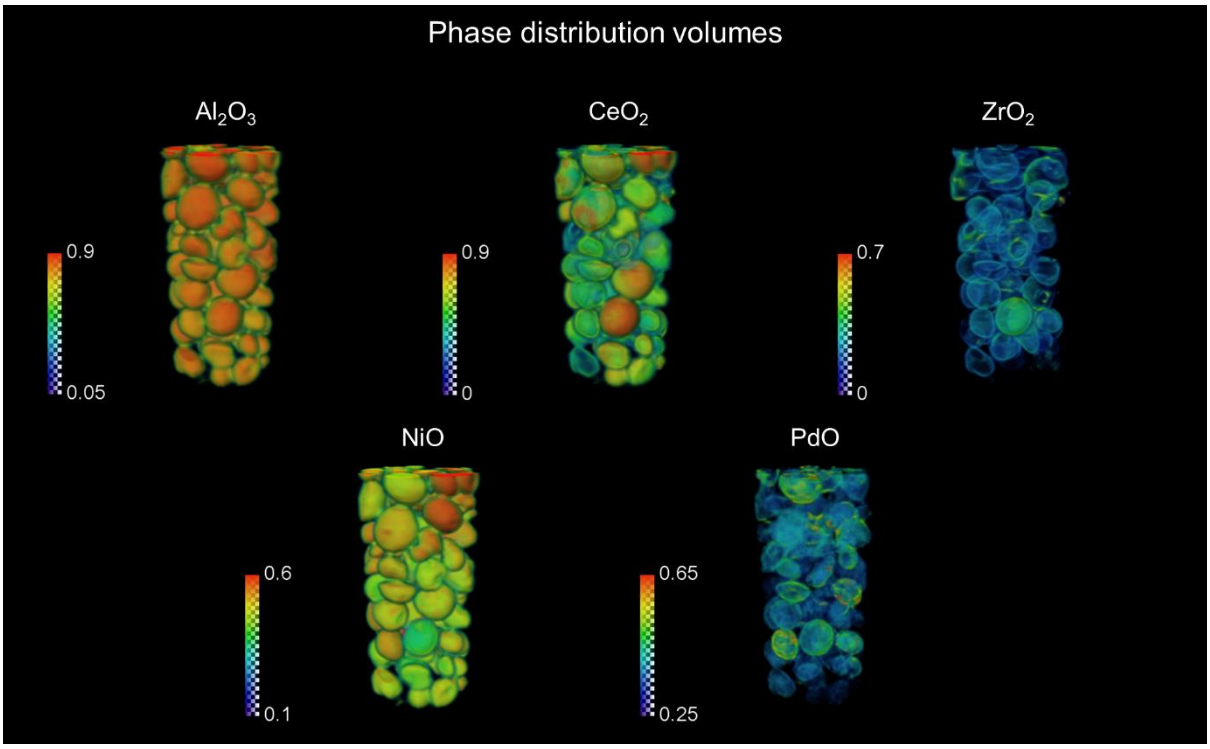

Supplementary Figure 6: Rietveld analysis of the 3D-XRD-CT data collected at ambient conditions. Volume rendering of the normalised Al_2O_3, CeO_2, ZrO_2, NiO and PdO scale factors data volume (phase distribution volumes). The values in the colorbar axes have been chosen to achieve the best possible contrast.

In Supplementary Figure 7, a (normalized) micro-CT image of the same catalyst is compared with the information obtained from the Rietveld analysis of the XRD-CT dataset acquired at the same z position. It can be seen that the absorption-contrast image cannot resolve the local physico-chemical heterogeneities of this complex Ni – Pd/ CeO_2 – ZrO_2/ Al_2O_3 catalyst. On the other hand, the Al_2O_3, NiO, CeO_2, ZrO_2 and PdO phase distribution maps also presented in Supplementary Figure 7 provide direct evidence that it is possible to obtain such information with an XRD-CT scan. These maps correspond to the normalised scale factors for the various phases present in the catalyst as obtained from the Rietveld analysis of this XRD-CT dataset.

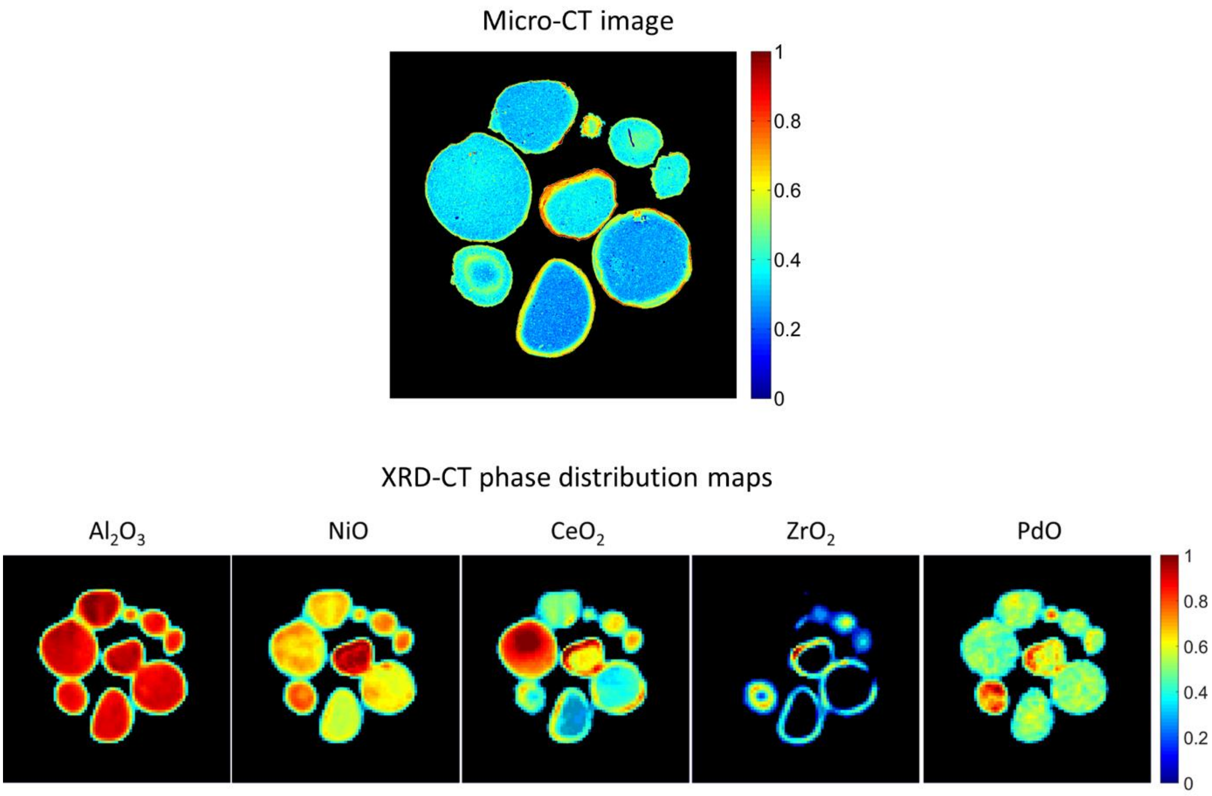

Supplementary Figure 7: Comparison of micro-CT data and XRD-CT data obtained at the z same position. Top: Micro-CT image. Bottom: The XRD-CT phase distribution maps correspond to the scale factors for the various phases as obtained from the Rietveld analysis.

High resolution XRD-CT measurement at ID15A, ESRF

A single catalyst particle was investigated with high resolution XRD-CT at beamline station ID15A of the ESRF using a 50 keV monochromatic X-ray beam focused to have a spot size of 1 μm x 1 μm with a Kb mirror system. 2D powder diffraction patterns were collected using the Pilatus3 X CdTe 2M area detector. The total acquisition time per point was 11 ms (exposure time of 10 ms and readout time of 1 ms). The tomographic measurements were made with 800 translation steps (translation step size of 1 μm) covering an 0 – 180 ° angular range with 600 angles. The detector calibration was performed using a CeO_2 NIST standard. Every 2D diffraction image was converted to a 1D powder diffraction pattern after applying an appropriate filter (i.e. 5 % trimmed mean filter) to remove outliers using pyFAI.[48, 49] The final XRD-CT images (i.e. reconstructed data volume) were reconstructed using the filtered back projection algorithm. The acquisition of the high resolution XRD-CT dataset lasted ca. 1.5 hours while the integration of the raw 2D diffraction patterns lasted ca. 34 hours and the Rietveld analysis of the 800 x 800 pixels XRD-CT images ca. 24 hours. These numbers clearly show that the rate limiting step in these experiments is related to data handling. The phase distribution maps of the various crystalline components present in the catalyst are shown in Supplementary Figure 8. It can be clearly seen that these results are in full agreement with the

lower resolution 3D-XRD-CT data. However, these high resolution data reveal more clearly that there are regions of high concentration of PdO (hotspots) showing that this phase is not well-dispersed over the catalyst particles.

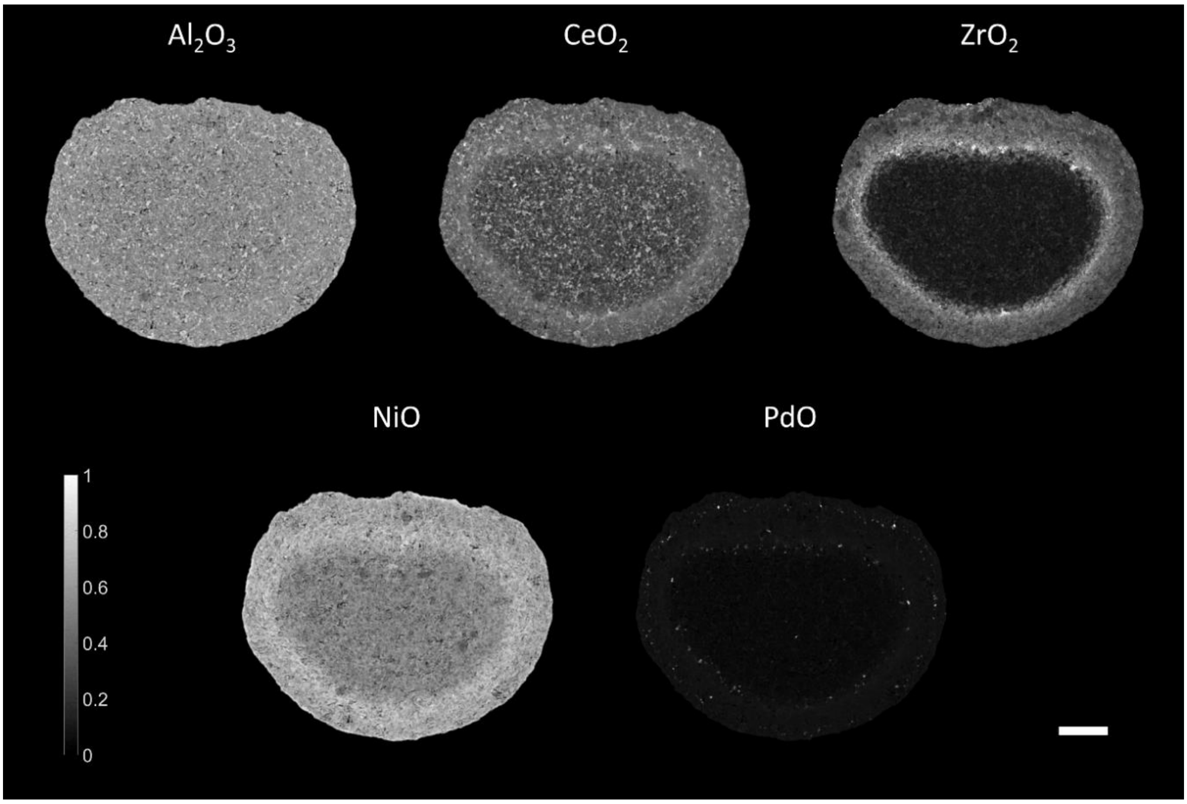

Supplementary Figure 8: Phase distribution maps of Al_2O_3, CeO_2, ZrO_2, NiO and PdO. The maps were derived from the Rietveld analysis of the single catalyst particle XRD-CT data. Scale bar corresponds to 0.1 mm.

The CeO_2 crystallite size and lattice parameter a maps shown in Supplementary Figure 9 are also in full agreement with the low resolution 3D-XRD-CT data and serve to prove that the results are directly reproducible.

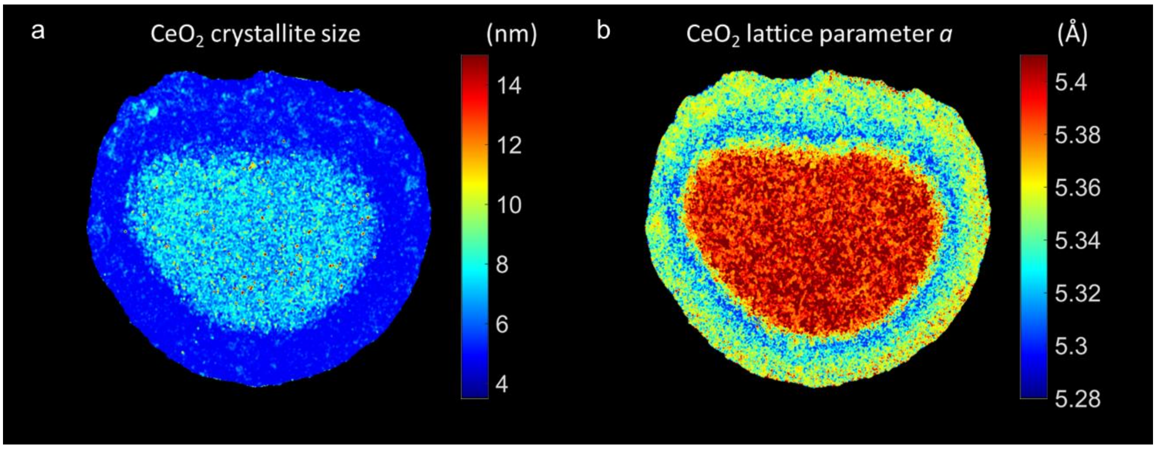

Supplementary Figure 9: Crystalline CeO_2 species. Panel A: Average crystallite size map of CeO_2 (colorbar axes in nm). Panel B: Map corresponding to the lattice parameter a of CeO_2 unit cell (colorbar axes in Å).

Supplementary note 5. Different chemical species of $Ce_xZr_yO_2$

In order to verify the existence of the four distinct crystalline $Ce_xZr_{1-x}O_2$ species, three summed diffraction patterns were exported from selected regions of interest. As it is shown in Supplementary Figure 10, three masks were created based on the results from the Rietveld analysis results presented in Figure 1 in the main paper and these masks correspond to a Ce rich $Ce_xZr_{1-x}O_2$ region (x >> 0), a Zr rich $Ce_xZr_{1-x}O_2$ region (x << 1) and another region where both phases are present (0 < x < 1). The three masks were applied to the reconstructed data volume (one at a time) and in each case the summed diffraction pattern was exported. These three diffraction patterns are plotted at the right side of Supplementary Figure 10 where a specific Q region is chosen (i.e. where the highest intensity CeO_2 and ZrO_2 peaks lie). It can be clearly seen that there is a peak shift to larger Q values for the ZrO_2 (green line in Supplementary Figure 10) in the ZrO_2 rich areas corresponding to a smaller unit cell (with respect to the $Ce_xZr_yO_2$ - blue line in Supplementary Figure 10). Similarly, there is a peak shift to smaller Q values for the CeO_2 (red line in Supplementary Figure 10) in the CeO_2 rich areas corresponding to a larger unit cell (with respect to the $Ce_xZr_yO_2$ - blue line in Supplementary Figure 10). Finally, it can also be readily observed that the broadening of the diffraction peaks (both for CeO_2 and ZrO_2) is higher in the region where both phases are present, implying the presence of smaller crystallites.

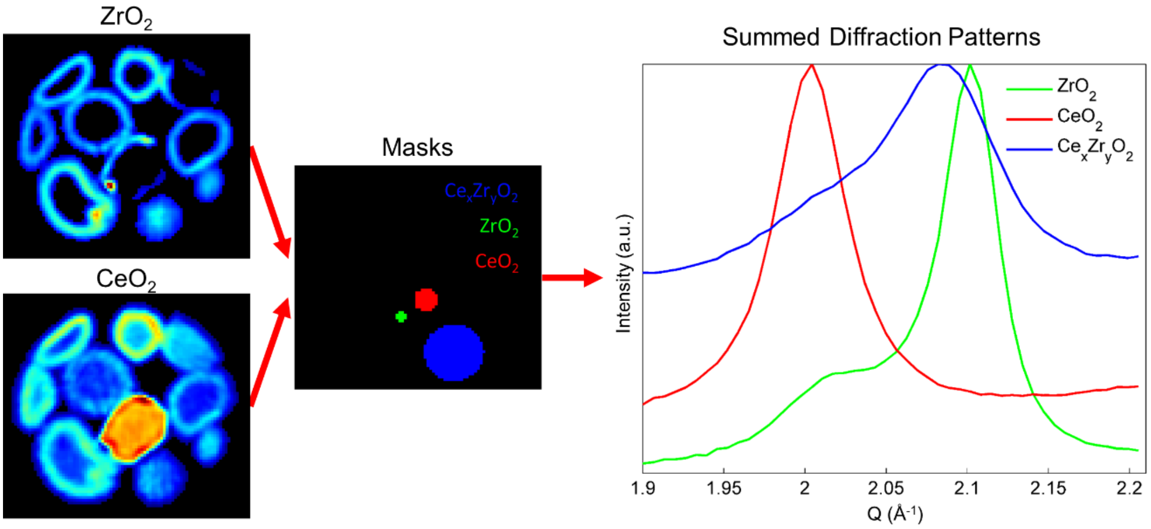

Supplementary Figure 10: Different chemical species of $Ce_xZr_yO_2$. Left: Maps corresponding to CeO_2 and ZrO_2. Middle: Masks created for three regions-of-interest (green, red and blue regions); these masks were applied to the XRD-CT data and in each case, the summed diffraction pattern was extracted from the data. Right: Regions of interest of three summed diffraction patterns exported from the three regions-of-interest. For comparison reasons, the diffraction patterns were normalized with respect to the highest intensity diffraction peak.

3D-XRD-CT at high temperature under He flow

The summed diffraction patterns of the 3D-XRD-CT data (30 XRD-CT scans) collected at 800 °C under He flow are shown at the right side of Supplementary Figure 11. For comparison reasons, the summed diffraction patterns of the 3D-XRD-CT data (30 XRD-CT scans) collected at room temperature are also presented at the left side of Supplementary Figure 11. It can be clearly seen that the intensity of main NiO diffraction peak (reflection (002)) has significantly decreased while the diffraction peak at ca. Q = 2.55 – 2.65 $Å^{-1}$ has not. Since there is a NiO diffraction peak in this Q region (reflection (111)), one would expect that the intensity of this peak would decrease too. However, this is not the case and this phenomenon was attributed to the formation and growth of the undesired $NiAl_2O_4$ phase (ICSD: 9554).[50, 51] It has been previously reported in literature that crystalline $NiAl_2O_4$ can be observed in Ni/Al_2O_3 catalysts calcined at temperatures above 600 °C.[52] It is no wonder then that the $NiAl_2O_4$ phase is seen to be present in the 10 wt. % Ni – 0.2 wt. Pd/ 10 wt. % CeO_2 – ZrO_2/ Al_2O_3 catalyst at 800 °C. The high intensity peak of the $NiAl_2O_4$ phase lies in the aforementioned Q region (reflection (311)). It should also be noted that no metallic crystalline Ni or Pd was observed in the 3D-XRD-CT data collected at 800 °C under He flow. This indicates that a reducing chemical environment (e.g. H_2) is essential in order to avoid the NiO to $NiAl_2O_4$ transition and reduce the NiO to the desired Ni phase as the formation and growth of the $NiAl_2O_4$ phase is a thermal effect and takes place even under inert chemical environment (He flow).

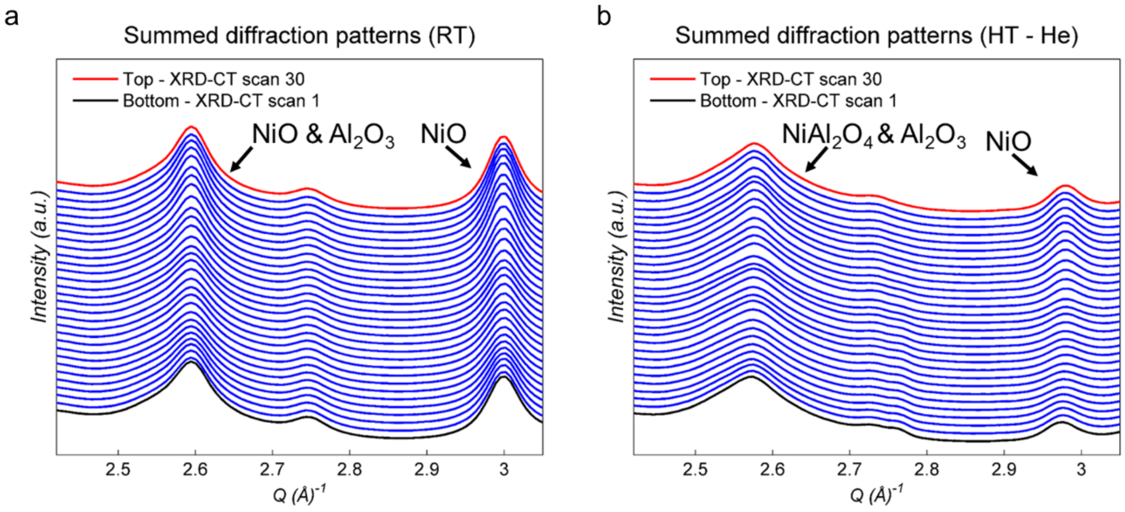

Supplementary Figure 11: Evolution of the Ni species during high temperature treatment. Panel a: The summed diffraction patterns of the 3D-XRD-CT data (30 XRD-CT scans) collected at room temperature. Panel b: The summed diffraction patterns of the 3D-XRD-CT data (30 XRD-CT scans) collected at 800 °C under He flow. In both cases, the main peak of NiO and the $NiAl_2O_4$ are indicated by the black arrows.

The phase distribution maps of certain crystalline phases of interest as obtained from the Rietveld analysis of the XRD-CT data collected at the bottom, middle and top of the sample (XRD-CT

scans 1, 15 and 30 respectively) are presented in Supplementary Figure 12. As discussed previously, the $NiAl_2O_4$ phase was also added to the model for the Rietveld analysis. The phase distribution maps shown in Supplementary Figure 12 correspond to the values of the scale factors as obtained from the Rietveld analysis of the respective XRD-CT data. These scale factors were then normalised with respect to the maximum value of each phase. These results show that there are not significant changes axially (i.e. as a function of z position) during the collection of this 3D-XRD-CT scan (ca. 1 h total acquisition time). A closer inspection of the results shown in Supplementary Figure 12 reveals that in the regions of the sample where the NiO signal is strong, the $NiAl_2O_4$ signal is weak and vice versa. More importantly though, the NiO phase is seen to be mainly present closer to the surface of the catalyst where the ZrO_2 phase (or Zr rich $Ce_xZr_yO_2$ phase to be more precise) is located. Such a characteristic example is provided in Supplementary Figure 12 (as indicated by the arrows) where two distinct regions are seen to be present in a catalyst particle: a) a NiO - Zr rich $Ce_xZr_yO_2$ layer near the surface of the catalyst and b) a $NiAl_2O_4$ rich core. In general, the $NiAl_2O_4$ phase is seen to predominantly form where the Zr rich $Ce_xZr_yO_2$ phase is absent. This is an important result as it provides indirect evidence that the role of the Zr rich $Ce_xZr_yO_2$ phase is to stabilize the NiO phase and suppress the formation of $NiAl_2O_4$ phase.

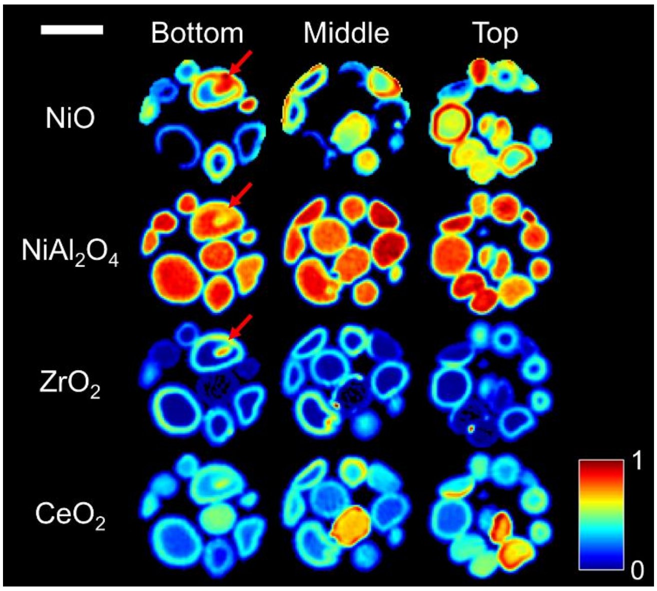

Supplementary Figure 12: High temperature treated catalyst bed. Phase distribution maps of NiO, $NiAl_2O_4$, ZrO_2 and CeO_2 obtained from the Rietveld analysis of the XRD-CT data collected at the bottom, middle and top of the sample (XRD-CT scans 1, 15 and 30 respectively). Red arrows indicate a region-of-interest showing that the CeO_2-ZrO_2 promoters suppress the formation of $NiAl_2O_4$. Color bar indicates intensity in arbitrary units. Scale bar corresponds to 1 mm.

In situ XRD-CT measurements during catalyst activation at ID15A, ESRF

In order to validate our assumption that the observed chemical gradient along the catalyst bed during the reduction process is a purely temporal phenomenon (i.e. for the Ni-containing phases), we performed a second diffraction experiment focusing on the behaviour of the catalyst during activation. The protocol for the catalyst pre-treatment was the same as in the 5D tomographic diffraction imaging experiment (see methods section). However, in this case, upon switching to 20 % H_2/He, 8 successive XRD maps of the whole catalyst bed were collected (z step size of 0.5 mm). The acquisition of each map lasted approximately 5 min. The phase distribution maps presented in Supplementary Figure 13 correspond to the (normalised) scale factors of NiO, $NiAl_2O_4$ and Ni (ICSD: 43397) as derived from the Rietveld analysis of these diffraction datasets. For each phase, the mean value of each map was calculated and then the maps were normalised with respect to the maximum of these values. The relative change in each phase is plotted on the right side of Supplementary Figure 13. It can be clearly seen that it takes more than 25 min (Ni plot in Supplementary Figure 13) for the catalyst to be fully reduced. It should be noted that the distance between the bottom and top of the catalyst bed was 5.5 mm which is more than 7× longer compared to the distance between the bottom and top of the 3D-XRDC-CT scan performed during the 5D tomographic diffraction imaging experiment (0.75 mm). Additionally, it can also be seen that near the middle of the catalyst bed, the concentration of each of these phases is almost identical. Specifically, the three rows in the middle of the maps presented in Supplementary Figure 13 correspond to a distance of 1 mm. This means that the XRD linescans in these three positions were acquired in ca. 1.5 min. It can be therefore concluded that Ni concentration the gradients observed in the 3D-XRD-CT data presented in Figures 4 and 5 due to a time effect and not z position.

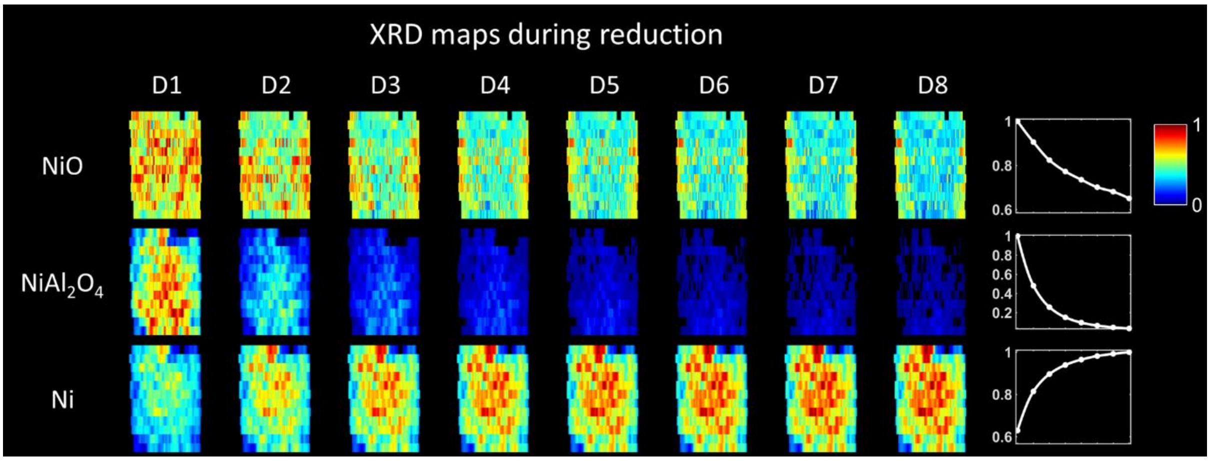

Supplementary Figure 13: Activation process of the catalyst. Phase distribution maps of NiO, $NiAl_2O_4$ and Ni corresponding to the normalized scale factors of these phases derived from the Rietveld analysis of the XRD maps collected during reduction. Right: Relative changes of each phase during the reduction process.

The phase distribution maps presented in Supplementary Figure 14 correspond to the scale factors of the NiO, $NiAl_2O_4$ and Ni phases as derived from the Rietveld analysis of the catalyst XRD-CT data collected at ambient conditions, at 800 °C under He flow and at 800 °C under 20 % H_2/He flow. The summed intensity of the scale factors over each XRD-CT scan was calculated and these values were then normalised with respect to the maximum value. These results are plotted at the right side of Supplementary Figure 14 and show the relative change of each crystalline phase during the experiment.

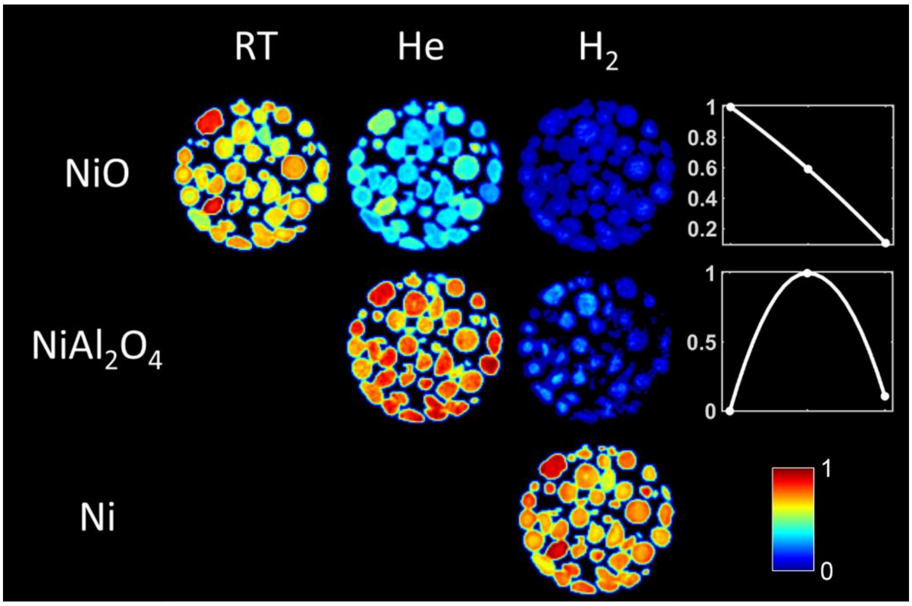

Supplementary Figure 14: Evolution of the crystalline Ni-containing species under different chemical environments. Phase distribution maps of NiO, $NiAl_2O_4$ and Ni corresponding to the scale factors of these phases as derived from the Rietveld analysis of the XRD-CT data collected at ambient conditions, at 800 °C under He flow and at 800 °C under 20 % H_2/He flow (i.e. data collected after the XRD maps). Right: Relative changes of each phase. Scale bar corresponds to 1 mm.

These XRD-CT scans were collected at the middle of the catalyst bed (middle rows in the maps shown in Supplementary Figure 13). It can be seen that the concentration of the NiO has decreased significantly (i.e. almost halved) during the temperature treatment under He and $NiAl_2O_4$ has formed. These results are in full agreement with the results obtained from the 5D tomographic diffraction imaging experiment showing also that the results are directly reproducible. It should be noted that a 3D-XRD-CT scan was near the middle of the catalyst bed was performed after the XRD maps (Supplementary Figure 13) under reducing conditions. The phase distribution maps of the NiO, $NiAl_2O_4$ and Ni phases as derived from the Rietveld analysis of the 3D-XRD-CT (10 XRD-CT datasets) are shown in Supplementary Figure 15. The 3D-XRD-CT scan covered approximately the same sample volume (i.e. the distance between the bottom and top of the 3D-XRDC-CT scan was 0.8 mm)

as the 3D-XRD-CT scan performed during the 5D tomographic diffraction imaging experiment. These results also serve to show that after ca. 40 min (as the 3D-XRD-CT scan was collected after the 8 XRD maps), there are no NiO/$NiAl_2O_4$/Ni concentration gradients along the bed. As shown also in Supplementary Figures 13 an 14, metallic Ni is the predominant Ni phase and the NiO and $NiAl_2O_4$ phases are present as traces.

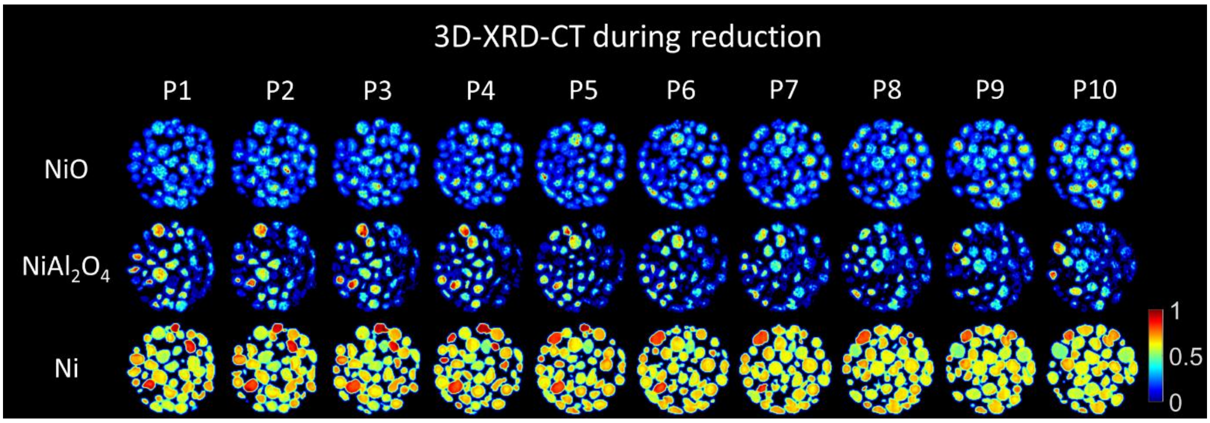

Supplementary Figure 15: 3D-XRD-CT results regarding the crystalline Ni-containing species during reduction. Phase distribution maps of NiO, $NiAl_2O_4$ and Ni corresponding to the scale factors of these phases as derived from the Rietveld analysis of the 3D-XRD-CT data collected at 800 °C under 20 % H_2/He flow (i.e. data collected after the XRD maps).

3D-XRD-CT at high temperature under 20 % O_2/He flow

At the left side of Supplementary Figure 16, the summed diffraction patterns of the 3D-XRD-CT data (30 XRD-CT scans) are shown. It can be seen that the metallic Ni peak is absent in all datasets and that only the NiO and $NiAl_2O_4$ phases can be observed. At the right side of Supplementary Figure 16, the summed diffraction patterns from the top, middle and bottom (XRD-CT scans 30, 15 and 1 respectively) of the sample volume probed during the high temperature 3D-XRD-CT measurement under 20 % O_2/He flow are shown. It can be observed that the intensity of the NiO peak decreases during the 3D-XRD-CT scan as it is gradually converted to $NiAl_2O_4$.

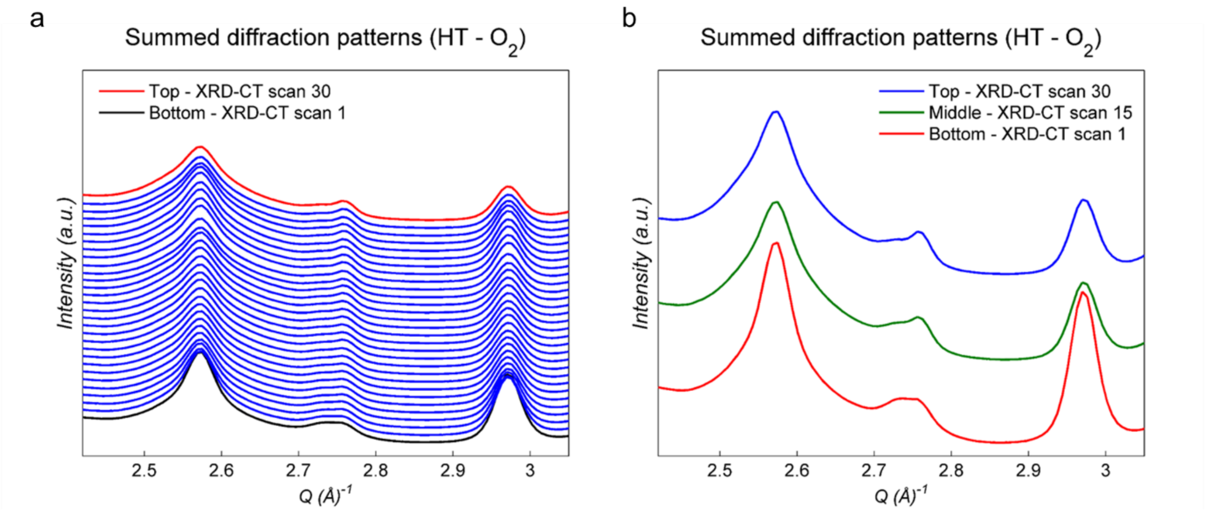

Supplementary Figure 16: Re-oxidation process. Panel a: The summed diffraction patterns of the 3D-XRD-CT data (30 XRD-CT scans) collected at 800 °C under 20 % O_2/He flow. Panel b: Summed diffraction patterns from the top, middle and bottom (XRD-CT scans 30, 15 and 1 respectively) of the sample volume probed during the high temperature 3D-XRD-CT measurement under 20 % O_2/He flow

Phase identification of graphite and Ni_xPd_y

On the left side of Supplementary Figure 17, a summed diffraction pattern from a region of interest of the last XRD-CT dataset collected during the operando POX experiment is presented. Phase identification showed that the diffraction peak observed at approximately Q = 1.81 $Å^{-1}$ (left side of Supplementary Figure 17) corresponds to the highest intensity peak (reflection (002)) of crystalline graphite ICSD: 76767.[1, 51, 53] On the right side of Supplementary Figure 17, a summed diffraction pattern from a region of interest of the second XRD-CT dataset collected under 20 % H_2/He flow (XRD-CT scan 4) is presented. Phase identification showed that this diffraction peak does not correspond to pure metallic Pd (ICSD: 52251) as it is found at ca. Q = 2.83 $Å^{-1}$ which is a high value for metallic Pd. This phase probably corresponds to Pd rich Ni_xPd_y (x<<y) alloy as the incorporation of Ni atom in the Pd unit cell will lead to a smaller unit cell which will generate the respective diffraction peak at higher Q values.[54, 55, 56, 57]

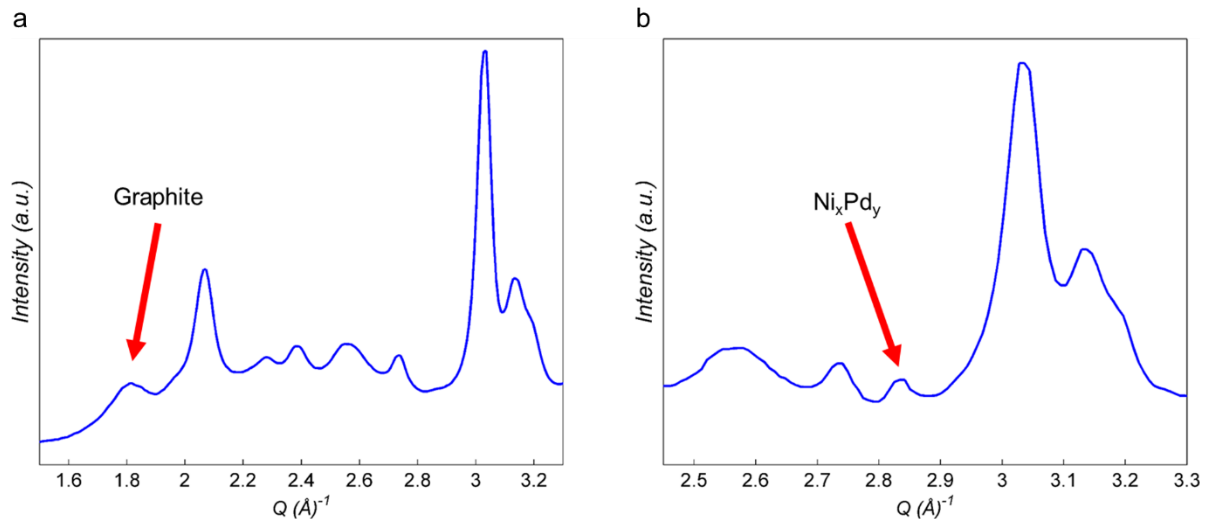

Supplementary Figure 17: Identification of the new phases formed during the 5D and redox experiments. Panel a: Summed diffraction pattern from a region of interest of the last XRD-CT dataset collected during the operando POX experiment demonstrating the highest intensity graphite peak (as indicated by the red arrow). Panel b: Summed diffraction pattern from a region of interest of the second XRD-CT dataset collected under 20 % H_2/He flow (XRD-CT scan 4) demonstrating the high intensity Pd peak (as indicated by the red arrow).

Mass spectrometry data during the in situ POX XRD-CT measurements

The mass spectrometry data acquired during the POX experiment are presented in Supplementary Figure 18, where the signals from specific masses of interest are shown and serve to prove that the catalyst was captured in its active state during the POX reaction. It can be clearly seen that upon switching to POX reaction conditions (i.e. region 1 in Supplementary Figure 18), the signal from masses 2 and 28 increase which correspond to fractions from the POX reaction products (i.e. H_2 and CO respectively). It can also be seen that the signal from masses 15, 17 and 44 (corresponding to CH_4, H_2O and CO_2 respectively) also increase and are present for the duration of the POX experiment. This means not only that CH_4 is not fully consumed (which is expected from the CH_4/O_2 molar ratio used) but that other side reactions take place too resulting in the formation of H_2O and CO_2. It can be clearly seen that the signal from all masses is stable for the duration of the POX experiment and no apparent deactivation of the catalyst was observed. However, as it is shown in Figure 8 in the main paper, upon switching to the POX reaction mixture a new phase formed which was identified as graphite (Supplementary Figure 17).

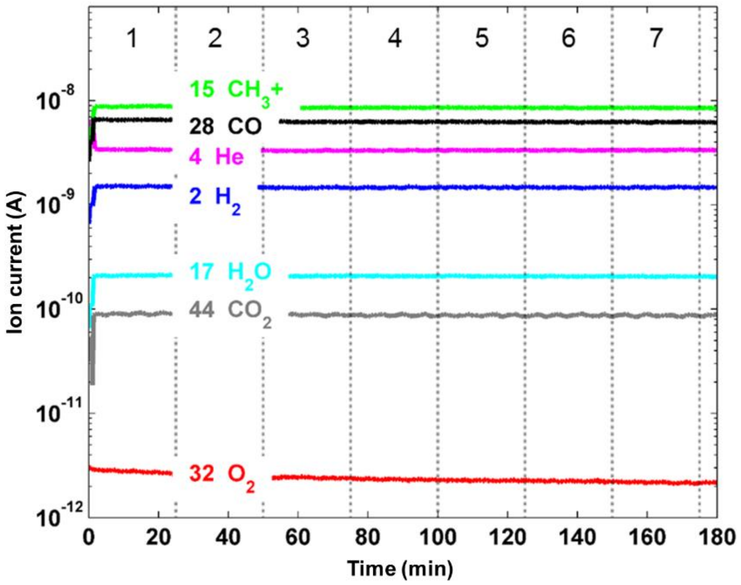

Supplementary Figure 18: Mass spectrometry results from the in situ POX XRD-CT experiment. Mass spectrometry data corresponding to the following m/z ratios (possible species and respective line colour in parentheses): 2 (H_2 - blue), 4 (He - magenta), 15 (CH_4+ - green), 17 (H_2O - cyan), 28 (CO – black), 32 (O_2 - red) and 44 (CO_2 - gray). The different regions (1-7) indicated correspond to the seven XRD-CT data collected under POX reaction conditions.

Laboratory POX catalytic activity experiment

The performance of the 10 wt. % Ni – 0.2 wt. Pd/ 10 wt. % CeO_2 – ZrO_2/ Al_2O_3 catalyst (500 mg weight loading) during POX was also investigated in the laboratory with quadrupole mass-spectrometric gas analysis measurements. The experimental protocol followed during this experiment is shown in the schematic presented in Supplementary Figure 19. Similarly to the in situ reactor cells used during the beamtime experiments, the outflow gasses were monitored by mass spectrometry using a Stanford Research Systems SRS QMS 300 mass spectrometric gas analyzer.

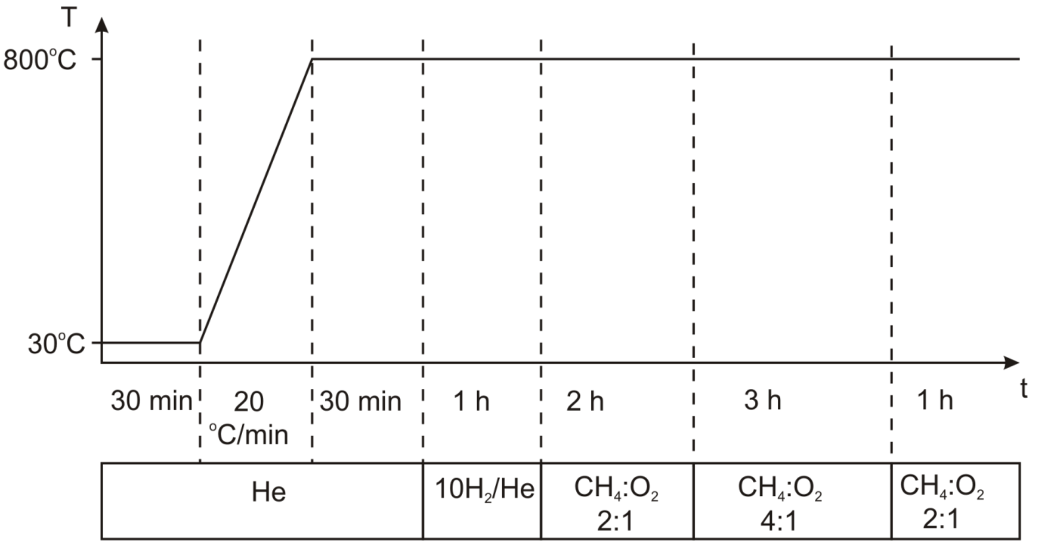

Supplementary Figure 19: Schematic diagram of the laboratory POX experiment with the 10 wt. % Ni – 0.2 wt. Pd/ 10 wt. % CeO_2 – ZrO_2/ Al_2O_3 catalyst

First, the catalyst sample was purged in the He flow (10 ml min^{-1}) for 30 min at the temperature of 30 °C. The temperature of the system was then increased to 800 °C with a ramp rate of 20 °C min^{-1} and a dwelling time of 30 min under He flow was applied before switching to reducing conditions. In this activation step, the catalyst was reduced under the flow 10 % H_2/He (H_2 : He = 1 : 9, total flow 100 ml min^{-1}) for 1 h. After this activation step, the POX reaction mixture of CH_4 : O_2 : He = 2 : 1 : 1.5 (total flow 240 ml min^{-1}) was fed to the reactor and the reaction products composition was analyzed during 2 h. After that, the O_2 feed rate was decreased and the reaction feed was switched to CH_4 : O_2 = 4 : 1 (CH_4 : O_2 : He = 2 : 0.5 : 1.5, total flow 220 ml min^{-1}). These operating conditions were maintained for 3h (GHSV = 440 ml min^{-1} mg^{-1}). As a final step, the ratio CH_4 : O_2 was returned to the initial one 2 : 1, in order to determine the variations of sample activity following its operation under the CH_4 rich conditions. The results from the catalytic activity measurements obtained during this experiment are presented in Supplementary Figure 20.

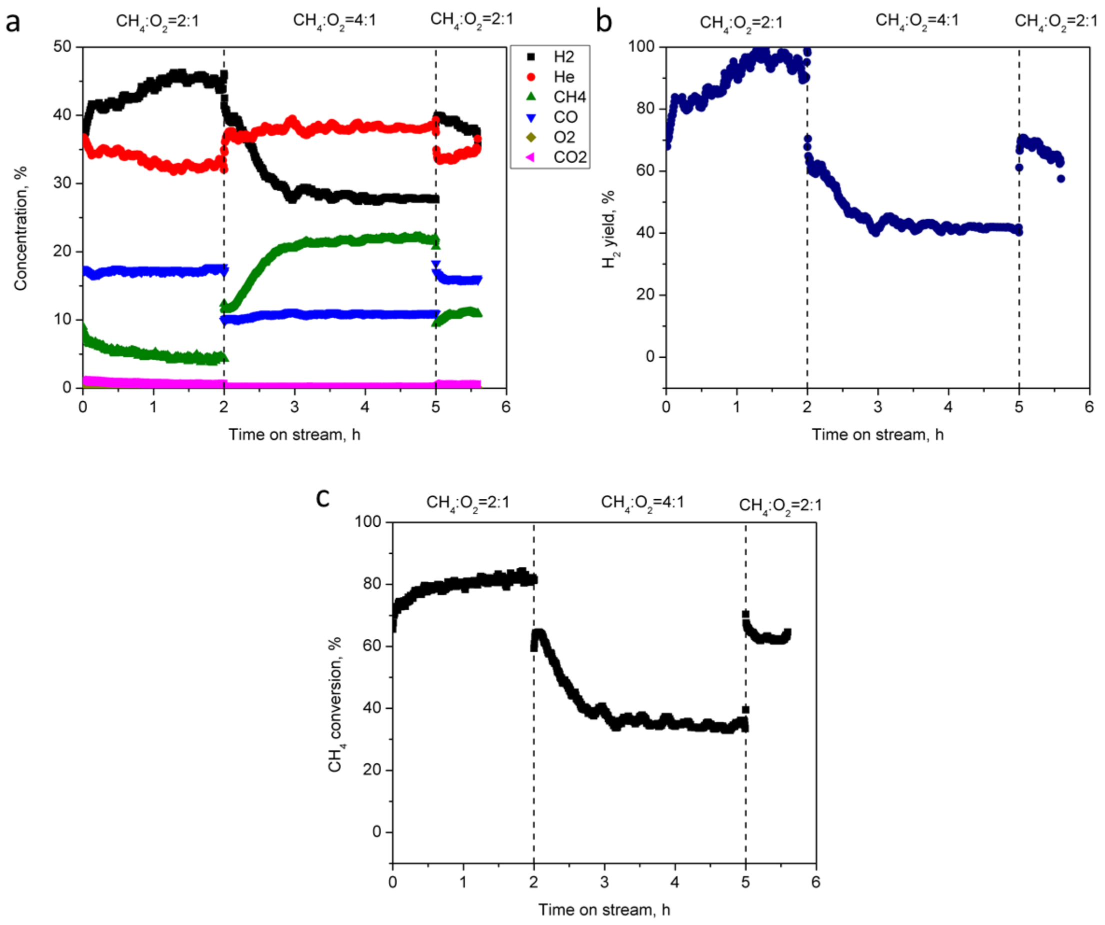

Supplementary Figure 20: Catalytic activity results from the laboratory POX experiment. Panel a: Concentration (%) of H_2, He, CH_4, CO, O_2, CO and CO_2 during the POX experiment. Panel b: Yield (%) of H_2 produced as a function of time. Panel c: CH_4 conversion (%) as a function of time.

As it is clearly shown in Panels A and B of Supplementary Figure 20, the performance of the catalyst is continuously increased during the first 2 h of the POX experiment. Specifically, the concentration of H_2 in the output stream is seen to rise while the concentration of CH_4 is decreasing. This is directly related to the activity of the catalyst as Panel C of Supplementary Figure 20 shows that the CH_4 conversion is continuously increasing during this time period, reaching a high of ca. 84 %. At the same time, we observe the H_2 yield reaches levels significantly higher than 90% (Panel B of Supplementary Figure 20). These results show that indeed this catalyst shows excellent results when the stoichiometric POX reaction ratio CH_4:O_2 ratio of 2:1 is used. However, it can be clearly seen that upon switching to the harsh reaction mixture (i.e. CH_4:O_2 ratio of 4:1), there is a drop in the catalyst performance. This is of course to be expected as in this CH_4 rich stream there is not enough O_2 to allow for its full conversion. During this 3 h time period, it can also be seen that CO_2 concentration is minimized for the exact same reasons. It is important to note though that the system is seen to stabilize after 1 h of operation under these harsh operating conditions and show constant

performance for the next 2 h of operation. This result is in full agreement with what we observed during the operando XRD-CT experiment, where the performance of the catalyst was seen to be stable for ca. 3 h. However, the most important results are related to the performance of the catalyst after this step, when the CH_4:O_2 ratio is switched back to 2:1. As it is clearly shown in all Panels of Supplementary Figure 20, the catalyst does not regain its initial performance. It can be seen that both the CH_4 conversion (ca. 65 % compared to >80 %) and the H_2 concentration are significantly lower (ca. 39 % compared to 45 %). However, it can also be seen that the catalyst has already started deactivating as both the H_2 concentration and H_2 yield show a decreasing trend. This result is very important as it is probably directly related to the formation and continuous growth of the crystalline graphite phase we observed during the operando XRD-CT experiment (CH_4:O_2 ratio of 4:1). It is therefore implied that this phase (and probably other non-crystalline C-containing species) is indeed detrimental to the long-term stability of this catalyst and is probably not removed when the CH_4:O_2 ratio is switched back to 2:1. Further studies could focus on optimizing a catalyst regeneration step but it should be mentioned that introducing an O_2 rich stream (in order to remove the carbonic species present in the catalyst) can also be harmful to the catalyst (e.g. by leading to sintering of the Ni particles as shown in Figure 9 in the main paper or by creating local hotspots due to the very exothermic nature of the reaction).

Scanning electron microscopy and elemental analysis

Cross-sectioned fresh and spent (i.e. after the laboratory POX experiment) catalyst particles were imaged and analysed using Scanning Electron Microscopy coupled with Enegry Dispersive X-ray Spectrometry (SEM-EDS). The morphological and elemental data were collected for the pre and post operation samples on a FEI Nova NanoSEM 450 scanning electron microscope (SEM) with an ultra-stable, high current Schottky field-emission source, fitted with an EDS detectors using an accelerating voltage of 20 kV.

The results from the fresh SEM/EDS measurements for the fresh Ni-Pd/CeO_2-ZrO_2/Al_2O_3 catalyst are shown in Supplementary Figures 21 and 22. In Supplementary Figure 21 it can be seen that two different scans were performed in the same area of the sample, taken at different magnification levels. The area of interest is highlighted (blue colour) at the top left section of Supplementary Figure 21. The SEM images reveal the presence of a thin layer near the surface of the catalyst particle. This layer is ca. 5 – 15 micron thick. The elemental maps clearly show that it is a layer rich in Zr and Ce. This result is in direct agreement with the XRD-CT data presented in this work.

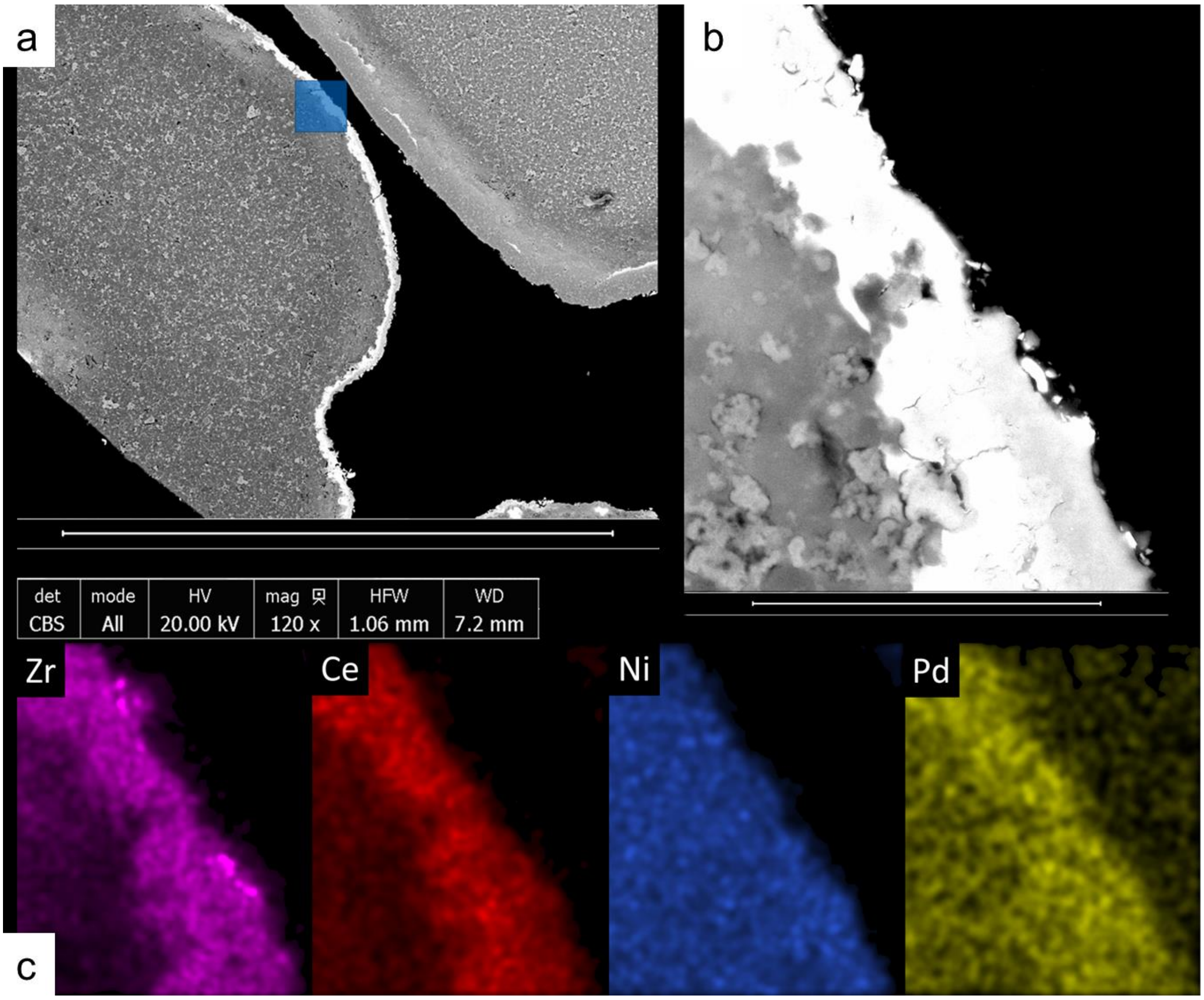

Supplementary Figure 21: SEM images and EDS mapping of the cross section of the fresh Ni-Pd/CeO_2-ZrO_2/Al_2O_3 catalyst. Imaging performed at two magnification levels (Panels a and b respectively). The magnified images (Panels B and C) correspond to region indicated by the blue rectangle on the top left (Panel A). Panel c: EDS maps for Zr, Ce, Ni and Pd. Scale bar on the left corresponds to 0.5 mm while the scale bar on the right corresponds to 0.02 mm.

This is further supported by the SEM/EDS images presented in Supplementary Figure 22 where it can be seen that the Zr species are predominantly present near the surface of the catalyst particles. This result is in direct agreement with the results obtained from the Rietveld analysis of the XRD-CT data that show the presence of a Zr rich $Ce_xZr_{1-x}O_2$ phase following an egg-shell distribution. In contrast to the Zr species, Ce species are seen to be present even near the core of the catalyst particles. This result is also in direct agreement with the XRD-CT results. However, it should be emphasized that the Rietveld analysis of the XRD-CT showed that the Ce rich $Ce_xZr_{1-x}O_2$ phase present near the core of the catalyst particles corresponds to larger crystallites compared to the surface species. It also allowed us to discriminate between two different $Ce_xZr_{1-x}O_2$ phases present near the surface of the catalyst particles (i.e. a Ce rich and a Zr rich one). This detailed physico-chemical information could have not be obtained with any other means.

In both Supplementary Figures 21 and 22, it can be seen that the Ni-containing species are present everywhere in the catalyst particles; a result which is also in full agreement with the results derived from the XRD-CT data. Finally, it can be seen that the signal of the Pd-containing species is higher near the surface of the catalyst particles. However, it can also be seen that there are regions of high concentration of this phase (i.e. hotspots). These results are also in direct agreement with the PdO (and Pd after reduction/activation) phase distribution maps and indicating that there is room for improvement regarding the catalyst design.

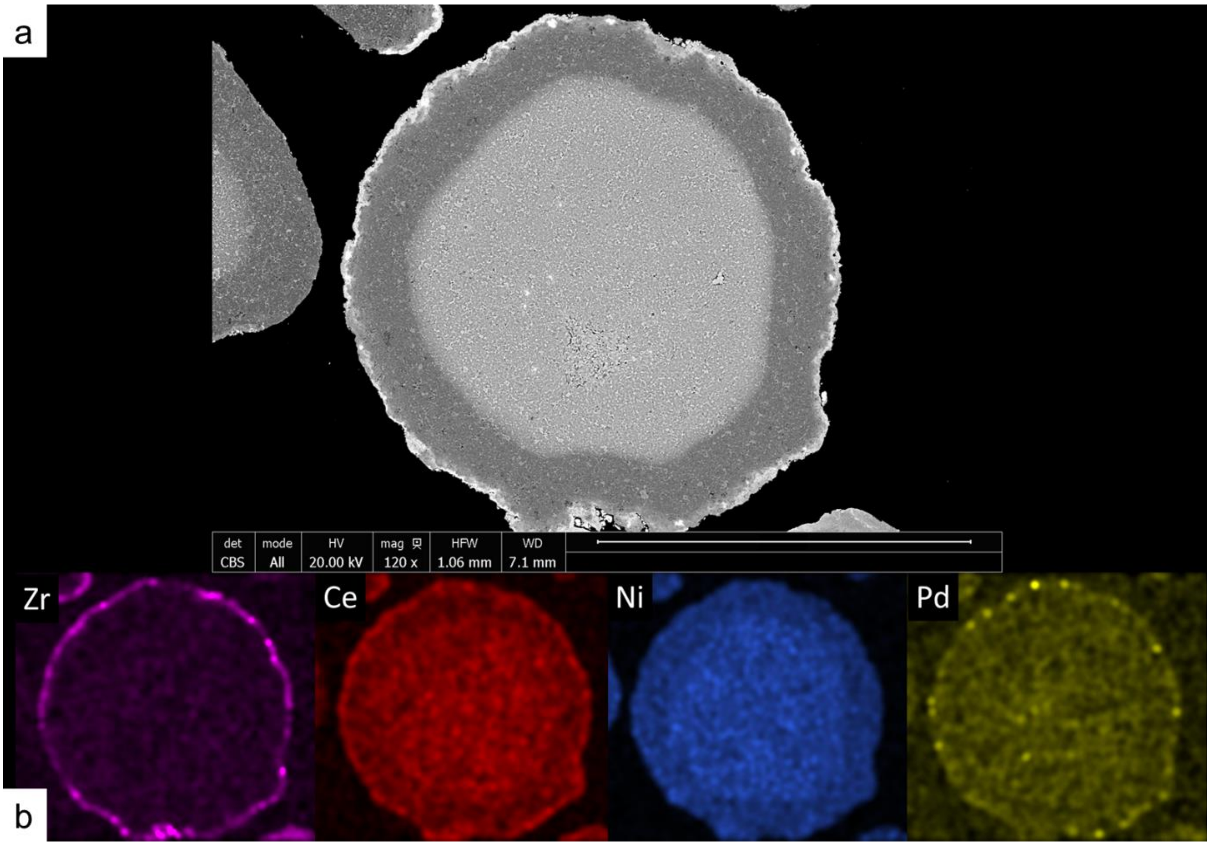

Supplementary Figure 22: SEM images and EDS mapping of the cross section of the fresh Ni-Pd/CeO_2-ZrO_2/Al_2O_3 catalyst. Panel a: SEM image. Panel b: EDS maps for Zr, Ce, Ni and Pd. Scale bar corresponds to 0.5 mm.

The results for the spent Ni-Pd/CeO_2-ZrO_2/Al_2O_3 catalyst after the laboratory POX experiment are shown in Supplementary Figures 23 and 24. It can be clearly seen in both Figures that the Zr species (Zr elemental maps) remained near the surface and did not migrate to inner core of the catalyst particles. Similar is the case for the Ce-containing species as there present everywhere in the catalyst particles (i.e. the Ce-containing species near the core of the catalyst particles did not migrate near the surface of the particles).

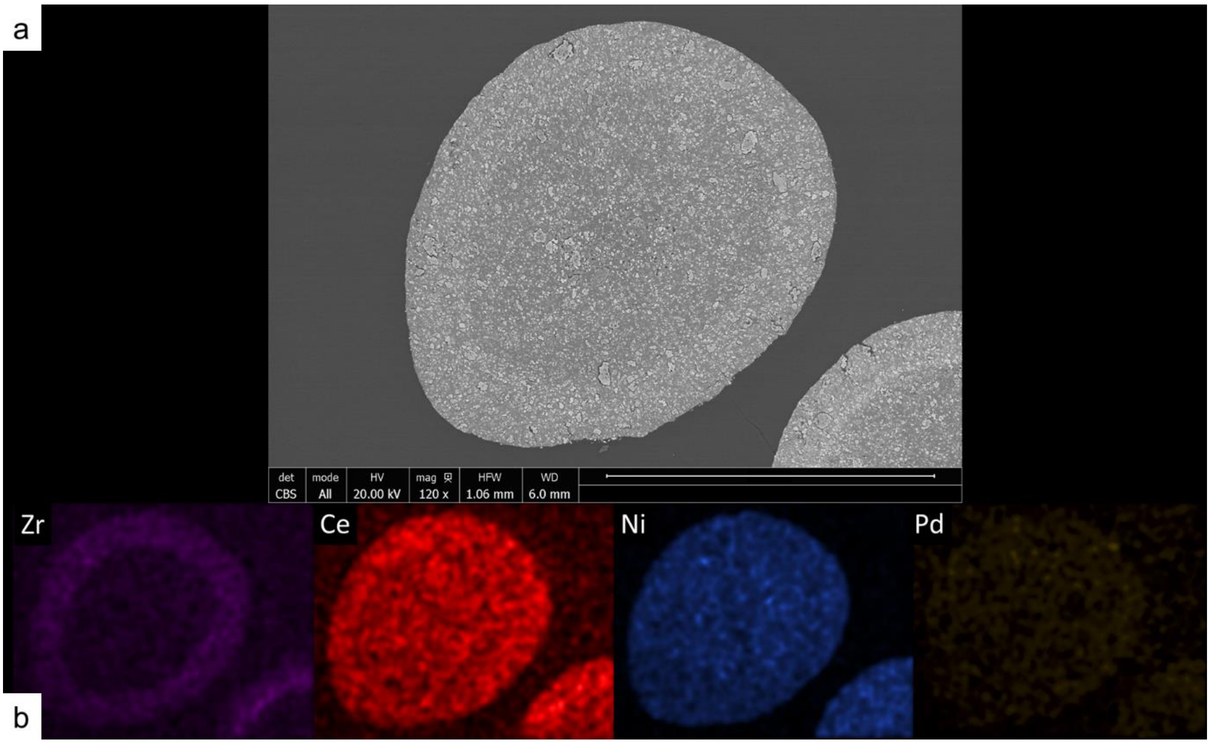

Supplementary Figure 23: SEM images and EDS mapping of the cross section of the spent Ni-Pd/CeO_2-ZrO_2/Al_2O_3 catalyst. Panel a: SEM image. Panel b: EDS maps for Zr, Ce, Ni and Pd. Scale bar corresponds to 0.5 mm.

The Ni species are also seen to be present everywhere in the catalyst particles; a result which is also in agreement with the XRD-CT data obtained under POX reaction conditions. The Pd signal was very weak in both scans so it is not easy to reach any definite conclusions. On the other hand, the SEM image presented in Supplementary Figure 24 shows there are minor cracks near the catalyst surface. However, this is not the case for the catalyst particle shown in Supplementary Figure 23. This phenomenon is probably associated with the position of the catalyst particles in the reactor. As this is an ex situ measurement of random particles taken from the spent catalyst, it is impossible to provide this information. This is another example showing why in situ and indeed operando characterization is essential in order to understand the structure-function relationships of a working catalyst and the reasons leading to their deactivation.

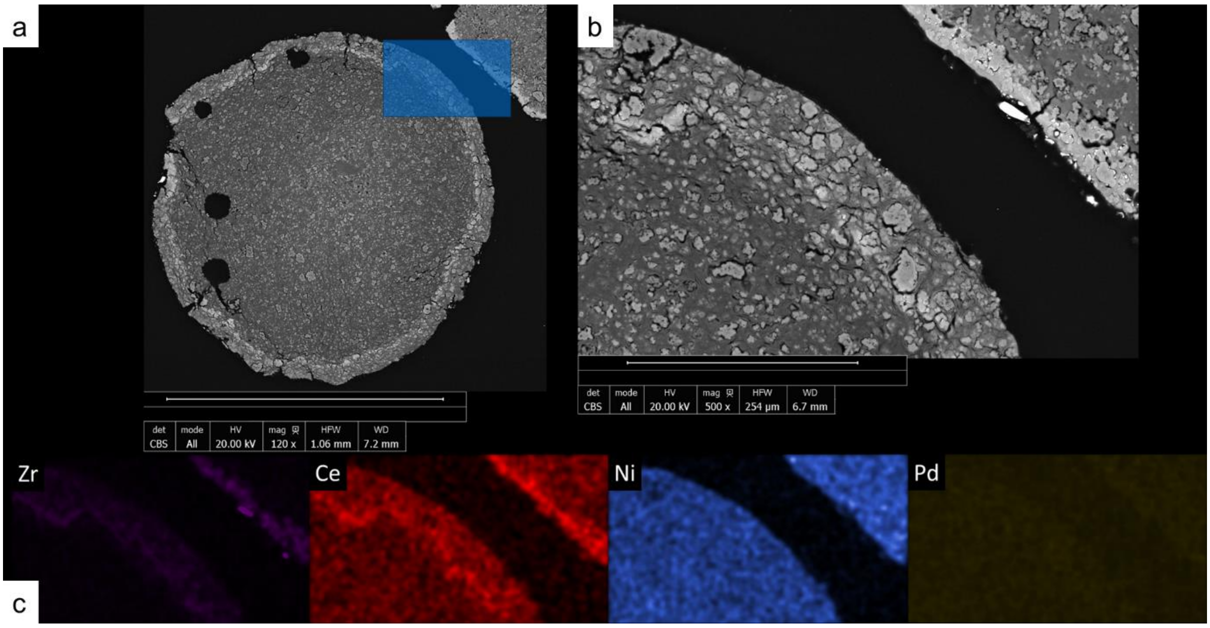

Supplementary Figure 24: SEM images and EDS mapping of the cross section of the spent Ni-Pd/CeO_2-ZrO_2/Al_2O_3 catalyst. Imaging performed at two magnification levels (Panels a and b respectively). The magnified images (Panels B and C) correspond to region indicated by the blue rectangle on the top left (Panel A). Panel c: EDS maps for Zr, Ce, Ni and Pd. Scale bar on the left corresponds to 0.5 mm while the scale bar on the right corresponds to 0.1 mm.

Thermogravimetric analysis

Thermogravimetric analysis (TGA) of the reduced and spent POX catalyst was performed by using a Q50 TA instruments. The temperature was increased from ambient to 950 °C with a ramp rate of 950 °C min^{-1}. Flow rates of 40 ml min^{-1} N_2 (balance) and 60 ml min^{-1} air (sample) were used during the experiment. The results are presented in Supplementary Figures 25 (thermogravinetric profile) and 26 (differential thermogravimetric profile - DT).

It can be seen that in the low-temperature range (T < 300 °C) there is a weight loss due to water desorption and other volatile species ($-\Delta m/m = 3\%$) for both the reduced and the spent POX catalyst. Above 300 °C, the reduced catalyst is seen to gain weight which is mainly due to the oxidation of metallic Ni to NiO. The weight is seen to stabilize upon reaching 600 °C and fully stabilize above 700 °C. On the contrary, for the spent catalyst, in the temperature range 350 – 660 °C there is a considerable weight loss ($-\Delta m/m = 32\ \%$) which corresponds to the burn-out of carbonaceous deposits on the catalyst. As shown in Supplementary Figure 26, the DT peak is at ca. 547 °C which is consistent with previous studies on Ni/Al_2O_3 catalysts claiming this carbonic species to be graphitic carbon.[58, 59]

No other peaks are observed strongly suggesting the presence of only one type of coke species present in the spent catalyst, which is the crystalline graphitic carbon identified by XRD-CT. In this temperature range no appreciable additional physical (i.e. sublimation, melting, evaporation) or chemical (dissociation, transformation due to the interaction with carbon) processes are expected, which could, otherwise, affect both the weight change and the heat effects during the DTA-TG measurements.

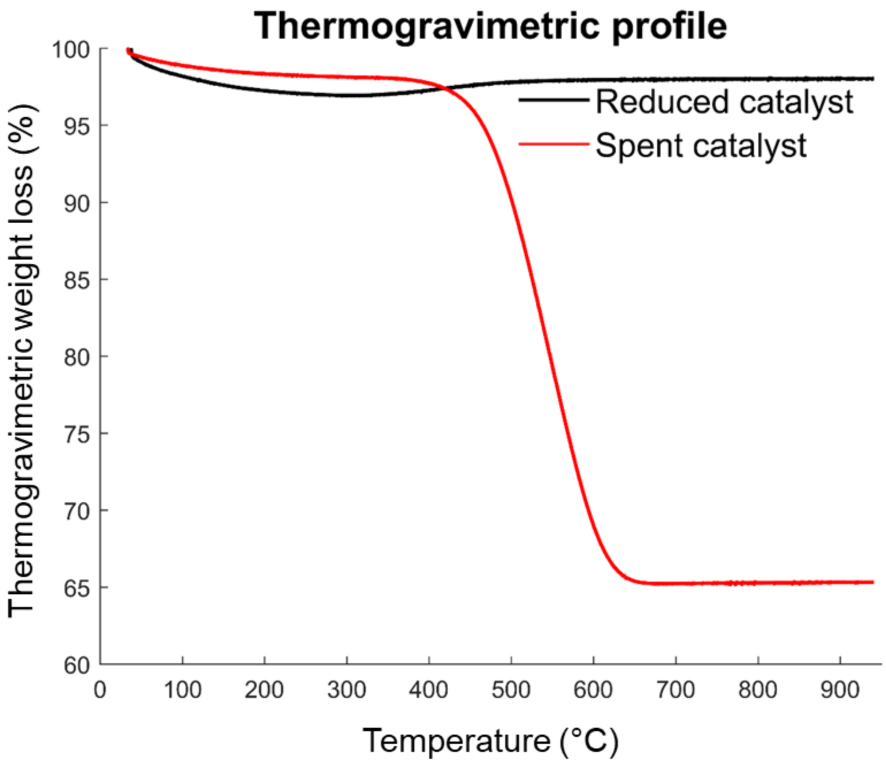

Supplementary Figure 25: Thermogravimetric profile of the reduced and spent Ni-Pd/CeO_2-ZrO_2/Al_2O_3 catalyst

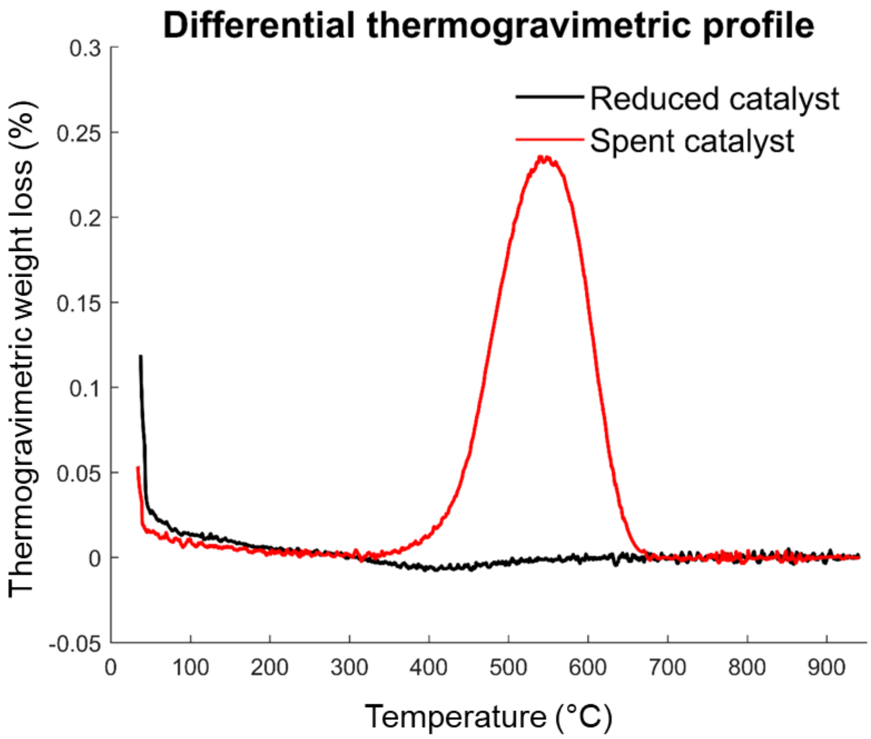

Supplementary Figure 26: Differential thermogravimetric profile of the reduced and spent Ni-Pd/CeO_2-ZrO_2/Al_2O_3 catalyst

Supplementary References

1. Harding, G., J. Kosanetzky, and U. Neitzel, X-ray diffraction computed tomography. Med. Phys., 1987. 14(4): p. 515-525.

2. Kleuker U, Suortti P, Weyrich W, Spanne P. Feasibility study of x-ray diffraction computed tomography for medical imaging. Phys. Med. Biol. 43, 2911-2923 (1998).

3. Stock SR, De Carlo F, Almer JD. High energy X-ray scattering tomography applied to bone. J. Struct. Biol. 161, 144-150 (2008).

4. Bleuet, P., et al., Probing the structure of heterogeneous diluted materials by diffraction tomography. Nat. Mater., 2008. 7(6): p. 468-472.

5. De Nolf W, Janssens K. Micro X-ray diffraction and fluorescence tomography for the study of multilayered automotive paints. Surf. Interface Anal. 42, 411-418 (2010).

6. Valentini L, Dalconi MC, Parisatto M, Cruciani G, Artioli G. Towards three-dimensional quantitative reconstruction of cement microstructure by X-ray diffraction microtomography J. App. Crystallogr. 44, 272-280 (2011).

7. Artioli G, et al. X-ray diffraction microtomography (XRD-CT), a novel tool for non-invasive mapping of phase development in cement materials. Anal. Bioanal. Chem. 397, 2131-2136 (2010).

8. Palancher H, Tucoulou R, Bleuet P, Bonnin A, Welcomme E, Cloetens P. Hard X-ray diffraction scanning tomography with sub-micrometre spatial resolution: application to an annealed γ-U0.85Mo0.15particle. J. App. Crystallogr. 44, 1111-1119 (2011).

9. Álvarez-Murga M, et al. Microstructural mapping of C60 phase transformation into disordered graphite at high pressure, using X-ray diffraction microtomography. J. App. Crystallogr. 44, 163-171 (2010).

10. Stock SR, Almer JD. Diffraction microcomputed tomography of an Al-matrix SiC-monofilament composite. J. App. Crystallogr. 45, 1077-1083 (2012).

11. Valentini L, Artioli G, Voltolini M, Dalconi MC, Jennings H. Multifractal Analysis of Calcium Silicate Hydrate (C-S-H) Mapped by X-ray Diffraction Microtomography. J. Am. Ceram. Soc. 95, 2647-2652 (2012).

12. Egan CK, et al. Non-invasive imaging of the crystalline structure within a human tooth. Acta Biomater. 9, 8337-8345 (2013).

13. Bonnin A, Wright JP, Tucoulou R, Palancher H. Impurity precipitation in atomized particles evidenced by nano x-ray diffraction computed tomography. Appl. Phys. Lett. 105, 084103 (2014).

14. Vanmeert F, Van der Snickt G, Janssens K. Plumbonacrite identified by X-ray powder diffraction tomography as a missing link during degradation of red lead in a Van Gogh painting. Angew. Chem. Int. Ed. 54, 3607-3610 (2015).

15. Wragg DS, O'Brien MG, Di Michiel M, Lonstad-Bleken F. Rietveld analysis of computed tomography and its application to methanol to olefin reactor beds. J. App. Crystallogr. 48, 1719-1728 (2015).

16. Jensen KMO, et al. X-Ray Diffraction Computed Tomography for Structural Analysis of Electrode Materials in Batteries. J. Electrochem. Soc. 162, A1310-A1314 (2015).

17. Frolich S, et al. Diffraction tomography and Rietveld refinement of a hydroxyapatite bone phantom. J. App. Crystallogr. 49, 103-109 (2016).

18. Beale AM, et al. X-ray physico-chemical imaging during activation of cobalt-based Fischer–Tropsch synthesis catalysts. Philos. T. Roy. Soc. A 376 (2018).

19. Voltolini M, et al. Understanding cement hydration at the microscale: new opportunities from `pencil-beam' synchrotron X-ray diffraction tomography. J. App. Crystallogr. 46, 142-152 (2013).

20. Beale AM, Jacques SD, Weckhuysen BM. Chemical imaging of catalytic solids with synchrotron radiation. Chem. Soc. Rev. 39, 4656-4672 (2010).

21. Grunwaldt JD, Schroer CG. Hard and soft X-ray microscopy and tomography in catalysis: bridging the different time and length scales. Chem. Soc. Rev. 39, 4741-4753 (2010).

22. Frenkel AI, Rodriguez JA, Chen JG. Synchrotron Techniques for In Situ Catalytic Studies: Capabilities, Challenges, and Opportunities. ACS Catal. 2, 2269-2280 (2012).

23. de Groot FMF, de Smit E, van Schooneveld MM, Aramburo LR, Weckhuysen BM. In-situ scanning transmission X-ray microscopy of catalytic solids and related nanomaterials. ChemPhysChem 11, 951-962 (2010).

24. Andrews JC, Weckhuysen BM. Hard X-ray spectroscopic nano-imaging of hierarchical functional materials at work. ChemPhysChem 14, 3655-3666 (2013).

25. Grunwaldt, J.-D., J.B. Wagner, and R.E. Dunin-Borkowski, Imaging Catalysts at Work: A Hierarchical Approach from the Macro- to the Meso- and Nano-scale. ChemCatChem, 2013. 5(1): p. 62-80.

26. Urakawa A. Trends and advances in Operando methodology. Curr. Opin. Chem. Eng. 12, 31-36 (2016).

27. Wang H, Wang F. In situ, operando measurements of rechargeable batteries. Curr. Opin. Chem. Eng. 13, 170-178 (2016).

28. Crumlin EJ, Liu Z, Bluhm H, Yang W, Guo J, Hussain Z. X-ray spectroscopy of energy materials under in situ/operando conditions. J. Electron Spectrosc. 200, 264-273 (2015).

29. Harks PPRML, Mulder FM, Notten PHL. In situ methods for Li-ion battery research: A review of recent developments. J. Power Sources 288, 92-105 (2015).

30. Deabate S, Gebel G, Huguet P, Morin A, Pourcelly G. 3 In situ and operando determination of the water content distribution in proton conducting membranes for fuel cells: a critical review. Energ. Environ. Sci. 5, 8824-8847 (2012).

31. Grey CP, Tarascon JM. Sustainability and in situ monitoring in battery development. Nat. Mater. 16, 45-56 (2017).

32. Jacques SD, et al. Dynamic X-ray diffraction computed tomography reveals real-time insight into catalyst active phase evolution. Angew. Chem. Int. Edit. 50, 10148-10152 (2011).

33. O'Brien MG, Jacques SDM, Di Michiel M, Barnes P, Weckhuysen BM, Beale AM. Active phase evolution in single Ni/Al2O3 methanation catalyst bodies studied in real time using combined μ-XRD-CT and μ-absorption-CT. Chem. Sci. 3, 509 (2012).

34. Beale AM, et al. Chemical imaging of the sulfur-induced deactivation of Cu/ZnO catalyst bodies. J. Catal. 314, 94-100 (2014).

35. Price SWT, Geraki K, Ignatyev K, Witte PT, Beale AM, Mosselmans JFW. In Situ Microfocus Chemical Computed Tomography of the Composition of a Single Catalyst Particle During Hydrogenation of Nitrobenzene in the Liquid Phase. Angew. Chem. Int. Edit. 127, 10024-10027 (2015).

36. Vamvakeros A, et al. Real time chemical imaging of a working catalytic membrane reactor during oxidative coupling of methane. Chem. Commun. 51, 12752-12755 (2015).

37. Vamvakeros A, et al. Interlaced X-ray diffraction computed tomography. J. App. Crystallogr. 49, 485-496 (2016).

38. Kovarik L, et al. Unraveling the Origin of Structural Disorder in High Temperature Transition Al2O3: Structure of θ-Al2O3. Chem. Mater. 27, 7042-7049 (2015).

39. Oh Y-S, Roh H-S, Jun K-W, Baek Y-S. A highly active catalyst, Ni/Ce–ZrO2/θ-Al2O3, for on-site H2 generation by steam methane reforming: pretreatment effect. Int. J. Hydrogen Energ. 28, 1387-1392 (2003).

40. Li C, Fu Y, Bian G, Hu T, Xie Y, Zhan J. CO2 Reforming of CH4 over Ni/CeO2-ZrO2-Al2O3 Prepared by Hydrothermal Synthesis Method. J. Nat. Gas Chem. 12, 167-177 (2003).

41. Roh H-S, Jun K-W, Park S-E. Methane-reforming reactions over Ni/Ce-ZrO2/θ-Al2O3 catalysts. Appl. Catal. A-Gen. 251, 275-283 (2003).

42. Cai X, Dong X, Lin W. Autothermal Reforming of Methane over Ni Catalysts Supported on CuO-ZrO2-CeO2-Al2O3. J. Nat. Gas Chem. 15, 122-126 (2006).

43. Cai X, Cai Y, Lin W. Autothermal reforming of methane over Ni catalysts supported over ZrO2-CeO2-Al2O3. J. Nat. Gas Chem. 17, 201-207 (2008).

44. de Freitas Silva T, Dias JAC, Maciel CG, Assaf JM. Ni/Al2O3 catalysts: effects of the promoters Ce, La and Zr on the methane steam and oxidative reforming reactions. Catal. Sci. Technol. 3, 635-643 (2013).

45. Hilli Y, Kinnunen NM, Suvanto M, Savimäki A, Kallinen K, Pakkanen TA. Preparation and characterization of Pd–Ni bimetallic catalysts for CO and C3H6 oxidation under stoichiometric conditions. Appl. Catal. A-Gen 497, 85-95 (2015).

46. Thompson P, Cox DE, Hastings JB. Rietveld refinement of Debye-Scherrer synchrotron X-ray data from Al2O3. J. App. Crystallogr. 20, 79-83 (1987).

47. TOPAS Version 5.0. Bruker AXS.

48. Vamvakeros A, et al. Removing multiple outliers and single-crystal artefacts from X-ray diffraction computed tomography data. J. App. Crystallogr. 48, 1943-1955 (2015).

49. Ashiotis G, et al. The fast azimuthal integration Python library: pyFAI. J. App. Crystallogr. 48, 510-519 (2015).

50. Boukha Z, Jiménez-González C, de Rivas B, González-Velasco JR, Gutiérrez-Ortiz JI, López-Fonseca R. Synthesis, characterisation and performance evaluation of spinel-derived Ni/Al2O3 catalysts for various methane reforming reactions. Appl. Catal. B-Environ. 158-159, 190-201 (2014).

51. Zhou L, Li L, Wei N, Li J, Basset JM. Effect of NiAl2O4 Formation on Ni/Al2O3 Stability during Dry Reforming of Methane. ChemCatChem 7, 2508-2516 (2015).

52. Li G, Hu L, Hill JM. Comparison of reducibility and stability of alumina-supported Ni catalysts prepared by impregnation and co-precipitation. Appl. Catal. A-Gen. 301, 16-24 (2006).

53. Zhang Q, Shen M, Wen J, Wang J, Fei Y. Partial oxidation of methane on Ni/CeO2-ZrO2/γ-Al2O3 prepared using different processes. J. Rare Earth 26, 347-351 (2008).

54. Mukainakano Y, Yoshida K, Okumura K, Kunimori K, Tomishige K. Catalytic performance and QXAFS analysis of Ni catalysts modified with Pd for oxidative steam reforming of methane. Catal. Today 132, 101-108 (2008).

55. Feng L, et al. Pd–Ni Alloy Nanoparticles as Effective Catalysts for Miyaura–Heck Coupling Reactions. J. Phys. Chem. C 119, 11511-11515 (2015).

56. Martínez de Yuso A, Le Meins J-M, Oumellal Y, Paul-Boncour V, Zlotea C, Matei Ghimbeu C. Facile and rapid one-pot microwave-assisted synthesis of Pd-Ni magnetic nanoalloys confined in mesoporous carbons. J. Nanopart. Res. 18, 380 (2016).

57. Bayat N, Rezaei M, Meshkani F. Hydrogen and carbon nanofibers synthesis by methane decomposition over Ni–Pd/Al2O3 catalyst. Int. J. Hydrogen Energ. 41, 5494-5503 (2016).

58. Guo J, Lou H, Zheng X. The deposition of coke from methane on a Ni/MgAl2O4 catalyst. Carbon 45, 1314-1321 (2007).

59. Zhou L, Li L, Wei N, Li J, Basset J-M. Effect of NiAl2O4 Formation on Ni/Al2O3 Stability during Dry Reforming of Methane. ChemCatChem 7, 2508-2516 (2015).
